# Mortalin Represents a Promising Therapeutic Target for Oral Cancers: Clinical Relevance and Experimental Evidence for the Activation of Akt/mTOR Signaling

**DOI:** 10.3390/cancers17172860

**Published:** 2025-08-30

**Authors:** Sosmitha Girisa, Mangala Hegde, Choudhary Harsha, Nafiseh Manteghi, Imliwati Longkumer, Gazi Naseem Ahmed, Munindra Narayan Baruah, Sunil C. Kaul, Renu Wadhwa, Ajaikumar B. Kunnumakkara

**Affiliations:** 1 Cancer Biology Laboratory, Department of Biosciences and Bioengineering, Indian Institute of Technology Guwahati (IITG), Guwahati 781039, Assam, India; sosmi176106101@iitg.ac.in (S.G.); mangala.hegde@rnd.iitg.ac.in (M.H.); harsha379@gmail.com (C.H.); nfs_manteghi@yahoo.com (N.M.); 2 North-East Cancer Hospital and Research Institute, Guwahati 781023, Assam, India; ilongkumer@yahoo.co.in (I.L.); drgnahmed@gmail.com (G.N.A.); munin_b@hotmail.com (M.N.B.); 3 AIST-INDIA DAILAB, National Institute of Advanced Industrial Science and Technology (AIST), Tsukuba 3058565, Japan; s-kaul@aist.go.jp

**Keywords:** oral cancer, mortalin, therapeutic target, Akt/mTOR pathway, apoptosis, therapy

## Abstract

Background: Oral cancer remains a significant global health concern due to its high incidence and mortality, as highlighted by GLOBOCAN 2022, and is characterized by poor survival rates despite available therapies. Therefore, there is an imperative need for developing novel therapeutic targets for this disease. Methods: This study investigates the oncogenic role of mortalin in oral cancer. We have used The Cancer Genome Atlas (TCGA) dataset, samples from North Eastern Region of India and tissue microarray to examine the expression of this gene/protein in patient samples. siRNA related knock down studies were carried out to determine the role of mortalin on oral cancer cell proliferation, survival, metastases, EMT, autophagy etc. Results: Analysis of TCGA dataset revealed increased mortalin expression in head and neck squamous cell carcinoma (HNSCC), which correlated with tumor grade and stage, and was associated with diminished overall survival. These findings were validated in oral cancer patient tissue samples obtained from the North East Region of India and oral cancer cell lines. Functional assays showed that mortalin knockdown via siRNA reduced cancer cell proliferation, migration, invasion, and angiogenesis while inducing apoptosis, disrupting mitochondrial membrane potential, and modulating autophagy. These effects were linked to altered expression of regulatory molecules, including p53, p21WAF1, cyclins, caspases, MMPs, Survivin, and components of the Akt/mTOR pathway, thereby alleviating key hallmarks of oral cancer. Conclusion: Collectively, these data support mortalin as a potential therapeutic target for oral cancer and warrant further studies for the development of mortalin-targeting drugs in both laboratory and clinical settings.

## Simple Summary

Oral cancer continues to be a significant health issue with low survival rates despite existing treatments. This study investigates a protein called mortalin and finds it to be highly expressed in oral cancer tissues, associated with poor patient survival outcomes. By lowering mortalin levels in oral cancer cells, researchers noted a decrease in cancer cell survival, proliferation, migration, and invasion, as well as an increase in cell death. The findings demonstrate that mortalin plays a crucial role in oral cancer progression and that its targeting could provide a promising new treatment approach. These results support future investigations aimed at developing more effective therapies for oral cancer.

## 1. Introduction

Oral cancer is a major public health concern, with high prevalence and mortality rates, particularly in South and Southeast Asian countries, including Bangladesh, India, and Sri Lanka [1,2]. According to GLOBOCAN, approximately 389,485 new cases and 188,230 deaths from lip and oral cavity cancers occurred worldwide in 2022 [3]. It can develop in various anatomical sites within the oral cavity and includes a range of histological subtypes [4]. Among these, oral squamous cell carcinoma (OSCC) is the most prevalent, comprising nearly 90% of all cases [5]. Other, less common forms include tumors of the salivary glands, soft tissue sarcomas, lymphomas of the oral cavity, and melanomas arising from the oral mucosa [6]. The key etiological factors for this malignancy include tobacco and alcohol usage, HPV infection, aging, poor oral hygiene, and poor diet [7,8]. Despite advancements in treatment methods, the five-year survival rate for patients remains below 60% due to late-stage diagnosis, limited effectiveness and adverse side effects of therapeutic agents, and tumor recurrence [9,10,11]. Therefore, it is crucial to explore potential therapeutic targets and identify safe, effective, and affordable treatments for this malignancy.

Mortalin, also known as GRP75/ HSPA9/mthsp70, is centrally involved in several biological processes, including chaperoning and protein folding, intracellular trafficking, and mitochondrial biogenesis [12]. Recent studies have demonstrated the pivotal role of mortalin in various cancers, elucidating its multifaceted functional implications [13,14,15,16,17,18,19,20,21,22,23]. Notably, overexpression of mortalin in diverse cancers of the brain, breast, bile duct, colon, gastric, liver, lung, ovary, pancreatic, prostate, and thyroid was correlated with poor prognosis and compromised survival in patients [13,15,16,17,18,19,20,21,22,23,24,25]. Upregulated mortalin was associated with enhanced cell survival and proliferation, as well as increased epithelial-to-mesenchymal transition (EMT), angiogenesis, invasion, and migration characteristics in cancer cells [14,22,26]. It has been reported that mortalin forms a complex with the tumor suppressor p53, a feature unique to cancer cells, thereby affecting the normal function of p53 [26,27]. The binding between mortalin and p53 inhibits the nuclear translocation and, consequently, the transcriptional activation function of p53. This impairment of p53 function prevents apoptosis and promotes the survival of cancer cells [27]. Normal cells lack the mortalin-p53 complex and display distinct subcellular localization of the two proteins [14,27]. Molecular studies have revealed the role of mortalin overexpression in promoting cancer cell proliferation, EMT, metastasis, stemness, and chemoresistance via multiple pathways, including Wnt/glycogen synthase kinase-3 beta/β-catenin, p53-p21WAF1, growth arrest and DNA-damage-inducible protein alpha, and mitogen-activated protein kinase (MEK)/extracellular signal-regulated kinase (ERK) [12,13,14,28,29,30,31]. Consistently, several studies have shown that suppression of mortalin attenuates oncogenic signaling pathways in experimental models of colon, ovarian, cervical, breast, and melanoma carcinomas [22,28,29,30]. Mechanistically, the inhibition of mortalin led to the induction of p21WAF1 and E-cadherin, along with the downregulation of signal transducer and activator of transcription (STAT)-3 signaling and inhibition of matrix metalloproteinases (MMP)-2, MMP-9, Vimentin, Slug, Twist, Vascular endothelial growth factor (VEGF), β-catenin, c-Myc, and cyclin D1 expression in cancer cells in various studies [12,14,26,28,29,30,31,32,33].

In the present study, we aimed to explore the role of mortalin in oral carcinogenesis and performed extensive analyses in clinically defined patients of this disease. Furthermore, oral cancer cell lines (possessing activated oncogenic signaling) and their mortalin-compromised derivatives were subjected to molecular analyses to explore the potential of mortalin as a therapeutic target for oral cancer.

## 2. Materials and Methods

### 2.1. Cell Culture and Reagents

The human oral cancer cell lines SAS (RRID: CVCL_6782) and HSC3 (RRID: CVCL_1288) were obtained from the Japanese Collection of Research Bioresources (JCRB) Cell Bank in Japan. The immortalized human keratinocytes, HaCaT (RRID: CVCL_0038), were obtained from the National Center for Cell Sciences (NCCS) in Pune, India. These cell lines were cultured in Dulbecco’s Modified Eagle Medium (DMEM) (12100-046, Invitrogen, Grand Island, NY, USA) supplemented with 10% fetal bovine serum (FBS) (10270-106, Gibco, Thermo Fisher Scientific, Grand Island, NY, USA) and 1× Penicillin-Streptomycin (15140122, Invitrogen), and they were maintained in a CO_2_ incubator at 37 °C with 5% CO_2_ and approximately 95% humidity.

### 2.2. In Silico Analysis

The Cancer Genome Atlas (TCGA) database, accessed using the TCGAbiolinks packages (version 2.32.0, DOI: 10.18129/B9.bioc.TCGAbiolinks) in R programming software (version 4.4.0), was utilized for transcriptomic data analysis [34,35] (https://www.R-project.org; accessed on 15 August 2023). TCGA data for 520 head and neck squamous cell carcinoma (HNSCC) patients and 44 normal samples were obtained for analysis. An a-priori power calculation was performed using an online danielsoper calculator (https://www.danielsoper.com/statcalc/calculator.aspx?id=47; accessed on 19 July 2025). Based on a Cohen’s d value of 0.5, a desired statistical power of 0.9, and a significance level of 0.05, the minimum required sample size was estimated to be 172. Overall survival (OS) probabilities of patients with HNSCC exhibiting high vs low levels of mortalin expression were determined using the KM plotter tool [36] (http://kmplot.com; accessed on 7 August 2023). The datasets obtained from the TCGA database were analyzed for the expression of mortalin across various categories, including stages, grades, tumor sizes, and nodal stages, within the HNSCC patient cohort. 

Quantitative data are presented as the mean ± standard deviation (SD) to reflect variability among biological replicates. For non-parametric data, results are expressed as median with interquartile range (IQR). For survival analysis, Kaplan–Meier survival curves were generated and compared using the log-rank test. Effect sizes were quantified using Cox proportional hazards models, and results are reported as hazard ratios (HRs) with corresponding 95% confidence intervals (CIs) to provide estimates of association and statistical precision.

### 2.3. Tissue Microarray

A tissue microarray comprising 208 paraffin-embedded oral tissues (normal and matched malignant specimens; 1 mm diameter and 5 µm thickness) from diverse patients was obtained from US Biomax, Inc., Derwood, MD, USA (Table S1).

### 2.4. Immunohistochemistry (IHC)

Mortalin expression was assessed in oral cancer tissues compared to adjacent normal oral epithelial tissues through IHC using an anti-mortalin antibody, biotinylated goat anti-mouse secondary antibody (Catalog# 62-6540, RRID AB_2533949, Invitrogen), and a peroxidase IHC Detection Kit (Cat. No. 36000; Invitrogen). The procedure followed the manufacturer’s protocol, which included successive steps of deparaffinization, rehydration, suppression of peroxidase, blocking, and incubation with primary and secondary antibodies. This was followed by treatment with an enzyme-conjugate solution, exposure to the DAB chromogen, and counterstaining with hematoxylin. After dehydration, the slide was sealed with a coverslip using DPX mountant and observed under a light microscope (BX43, Olympus, Tokyo, Japan). Tissues showing brown staining were considered positive for mortalin expression. 

Scoring of the IHC stain was evaluated based on both the intensity of staining and the percentage of positively stained tumor cells. Staining intensity was scored on a scale of 1 to 3, where 1 = weak, 2 = moderate, and 3 = strong. The percentage of positive cells was categorized into the following groups: <10%, 10–25%, 25–50%, 50–75%, and >75%. These percentages are scored as 0, 1+, 2+, 3+, and 4+, respectively. The total expression score was calculated by multiplying the intensity (I) score and the percentage of positive area (I) of the stain (Table S2).

### 2.5. Real-Time PCR

The mRNA expression of mortalin was determined in oral cancer patient samples collected from North-East Cancer Hospital and Research Institute (NECHRI), Jorabat, Assam, India (Table S3). Total RNA was isolated from tissue samples using TRI reagent, followed by cDNA synthesis with the High-Capacity cDNA Reverse Transcription Kit from Life Technologies, Invitrogen. RT-qPCR was performed using QuantStudio 5 software (Applied Biosystems, Waltham, MA, USA, Thermo Fisher Scientific). Glyceraldehyde 3-phosphate dehydrogenase (GAPDH) served as a standard gene to normalize the expression of the target gene. The primers for mortalin (forward sequence: AGCTGGAATGGCCTTAGTCAT; reverse sequence: CAGGAGTTGGTAGTACCCAAATC) were obtained from Integrated DNA Technologies (IDT), Inc., Coralville, IA, USA. A similar protocol was followed for determining the mRNA expression of mortalin in human normal and oral cancer cells.

### 2.6. SiRNA-Mediated Knockdown

Small interfering RNA (siRNA) targeting mortalin was obtained from Sigma Merck, St. Louis, MI, USA (siRNA ID- SASI_Hs01_00216923). Briefly, oral cancer cells were plated (at a density of 2 × 10^5^ cells per well) in a six-well plate. Upon attaining 70% confluency, the cells were treated with lipid-RNA complexes prepared in incomplete Opti-MEM medium, which comprised either 5–50 pM siRNA specific to mortalin or scrambled siRNA, along with the transfection reagent Lipofectamine RNAiMAX (Cat No. 13778150, Invitrogen), prepared following the manufacturer's instructions [37]. After 48 h of incubation, cells were harvested for subsequent analyses. Cells transfected with mortalin-specific siRNA were designated as the knockdown group (referred to as siRNA), while those transfected with scrambled siRNA served as the control group (referred to as Control). The knockdown of mortalin was confirmed by Western blot analysis.

### 2.7. Short-Term Cell Viability Assays

Cell proliferation was evaluated using the 3-[4,5-dimethylthiazol-2-yl]-2,5-diphenyl-2,5-diphenyl tetrazolium bromide (MTT- M6494, Invitrogen, Life Technologies, Carlsbad, CA, USA) assay following the manufacturer’s guidelines. Cells (2000/well of 96-well plate) were incubated at 37 °C for 24 h, followed by treatments (0–72 h, as indicated). The cells were incubated with MTT solution (5 mg/mL) for 2 h. The culture medium was then replaced with 100 μL DMSO (Code 1.16743.0521, Merck, Darmstadt, Germany), followed by 1 h incubation in the dark. The absorbance was measured at 570 nm using a SpectraMax iD3 microplate reader (Molecular Devices, San Jose, CA, USA). Data was calculated as the percentage inhibition of cell proliferation compared to the control. Viability of the control and mortalin-compromised cells was also determined by propidium iodide (PI—a fluorescent stain that intercalates with exposed nucleic acids, emitting red fluorescence)-based flow cytometry assay using FACS Celesta, BD Biosciences, Paramus, NJ, USA. 

### 2.8. Long-Term Cell Viability Assay

The long-term effects of siRNA were evaluated using the colony-forming assay. Cells were seeded at a low concentration (1–2 thousand cells/well in a 6-well plate), exposed to a single treatment with a low dose of siRNA for 24 h, followed by culture for 10–15 days with regular medium changes every two days. At the end of the experiment, the medium was discarded, and the colonies were fixed with chilled ethanol for 1 h. They were then stained with crystal violet (*w*/*v*, 0.01%, Cat No: 548-6209; Sisco Research Laboratories, Mumbai, India) for 15 min, followed by washing with PBS. Plates were dried overnight. Images were captured, and the cell colonies were scanned and analyzed using ImageJ software, version 1.54d (RRID: SCR_003070), developed by the National Institutes of Health (NIH) and the Laboratory for Optical and Computational Instrumentation (LOCI) at the University of Wisconsin. The survival fraction of the samples was then calculated as described earlier [38].

### 2.9. Cell Cycle Analysis

The control and siRNA-treated cells were collected and fixed with chilled 75% ethanol at −20 °C for 1 h. The fixed cells were incubated with PI/RNase (Cat No. A35126, Invitrogen) for 20 min in the dark and analyzed using a FACS Celesta flow cytometer from BD Biosciences. The percentage of cells in each cell cycle phase was determined using FCS Express software, version 5.0.74.0 (RRID: SCR_016431, De Novo Software, Pasadena, CA, USA). 

### 2.10. Annexin VAssay

The Annexin V assay was conducted to evaluate apoptosis in control and mortalin-knockdown oral cancer cells using the Annexin V apoptosis kit (RRID: AB_2575598, eBioscience, Invitrogen) following the manufacturer's procedures. Briefly, the cells (1 × 10^5^/well in a 6-well plate) were harvested, incubated with Annexin V, and analyzed using a FACS Celesta flow cytometer (from BD Biosciences) with BD FACSDiva software, version 8.0.1.1 (RRID: SCR_001456).

### 2.11. JC-1 Assay

Mitochondrial integrity in control and treated cells was determined by mitochondrial membrane potential-based JC-1 staining. The cells were plated (1 × 10^5^ cells/well) in a 6-well plate and treated with either control or siRNA for 48 h. The cells were collected by trypsinization, followed by 7 min of centrifugation at 125× *g*. The cell pellets were resuspended in PBS and centrifuged again under the same conditions. 5,5,6,6′-tetrachloro-1,1′,3,3′tetraethylbenzimidazoylcarbocyanine iodide dye (JC-1) was added to all samples, excluding the unstained control, and was incubated for 45 min. Subsequently, the samples were centrifuged, and the pellets were resuspended in PBS. The mitochondrial membrane potential was then analyzed using a FACS Celesta flow cytometer from BD Biosciences. Carbonyl cyanide m-chlorophenyl hydrazone (CCCP) dye was used as a positive reference (M34152, MitoProbe JC-1 Kit, Invitrogen).

### 2.12. Cell Migration Assay

The migration capacity of control and treated cells was determined using the wound-scratch assay. Cells (5 × 10^5^ cells/well in a 6-well plate) were seeded, allowed to settle overnight, and incubated until a uniform monolayer was formed. The cells were then serum-starved for 8 h. A scratch was made on the monolayer using a microtip (200 μL). Cells were rinsed with PBS to remove floating cells, incubated at 37 °C, and monitored every 6 h under the microscope. Images were captured at various time points using an inverted Nikon TS100 microscope and analyzed with ImageJ software; version 1.54d (RRID: SCR_003070, NIH & LOCI, University of Wisconsin, University of Wisconsin, WI, USA). 

### 2.13. Invasion Assay

The effect of mortalin-siRNA on the invasion capacity of cells was examined using a Boyden chamber assay, as described earlier [31,39]. Control and treated cells were plated at a concentration of 5 × 10^4^ cells per well (serum-starved cells) onto 8 μm pore transwell inserts (Corning, Corning, NY, USA), which were precoated with Matrigel and placed in wells of a 24-well plate. The media beneath the transwell inserts contained 30% FBS as a chemoattractant. Following a 24 h incubation, the migrated cells were fixed with formaldehyde (4%), stained with crystal violet solution (0.01% (*w*/*v*)), and captured images were taken using an inverted Nikon Eclipse TS100 microscope, and the invasion percentage was calculated using Image J software; version 1.54d (RRID: SCR_003070, NIH & LOCI, University of Wisconsin).

### 2.14. Immunocytochemistry (ICC)

Cells were plated onto coverslips inserted in a 12-well dish. Control and treated cells were subjected to immunostaining using antibodies to proteins involved in specific pathways, including autophagy (LC3B, Cat. No. L10382, Thermo Fisher Scientific) and EMT (Snail-NL557 and vimentin-NL493, Cat. No. SC026, R&D Systems, Minneapolis, MN, USA). Cells were fixed with formaldehyde, permeabilized, and blocked with bovine serum albumin (2% BSA), followed by primary antibody incubation for 24 h, rinsed with TPBS (PBS containing Triton-X-100 (0.1%) three times for 10 min each, and then treated with secondary antibody conjugated with Alexa Fluor 594 (Invitrogen). The stained cells were washed with TPBS and counterstained with 4′,6-diamidino-2-phenylindole (DAPI) and mounted onto a microscope glass slide with DPX solution (Rankem, Avantor Performance Materials India Limited, Gurgaon, India). Images were captured using an upright BX43 microscope (from Olympus). 

### 2.15. Western Blot Analysis

Western blotting was conducted as described previously [37,38]. Briefly, the transfected cells (3 × 10^5^ cells/well in a six-well plate) were harvested as whole cell lysates by scraping after the addition of lysis buffer (20 mM HEPES, 250 mM NaCl, 2 mM EDTA, 1 mM DTT, Triton X-100 (0.1% *v*/*v*), 2 μg/mL aprotinin, 1 mM PMSF, and 2 μg/mL leupeptin hemisulfate), followed by centrifugation (Heraeus, Megafuge 16R, Thermo Scientific). The supernatant was used as the whole-cell lysate, and protein concentration was quantified using the Bradford assay. Protein (10–20 μg) was resolved by SDS-PAGE and transferred onto a nitrocellulose membrane using a semi-dry transfer machine (Trans-Blot Turb, Bio-Rad, Hercules, CA, USA). Following this, the membrane was blocked with 2% BSA, incubated with primary antibodies (Table S4) for respective proteins for 24 h, treated with secondary antibody-horseradish peroxidase (HRP) conjugated, and blots were visualized with a ChemiDo XRS System from Bio-Rad, using Clarity Western ECL Substrate solution (Cat. No. 1705061; Bio-Rad). GAPDH was used as the loading reference, and densitometry was performed using Image Lab, version 6.1.

### 2.16. Statistical Analysis

Statistical analysis for cell line studies was performed using Student’s *t*-test, and results were expressed as the mean ± SD. Statistical analyses for tissue-related data were conducted using R (version 4.2.2) and GraphPad Prism (version 9.0). For comparisons between normal and tumor tissue samples from The Cancer Genome Atlas (TCGA), data distribution was first assessed using the Shapiro–Wilk test for normality and visual inspection of density and Q-Q plots. As several gene expression datasets deviated from a Gaussian distribution, non-parametric Mann–Whitney U tests were applied for two-group comparisons. This approach was chosen to account for the biological heterogeneity and non-normal distribution typically observed in transcriptomic datasets. All *p*-values are two-tailed, and significance was represented in *p*-values < 0.001 (*** highly significant), <0.01 (** very significant), <0.05 (* significant), and >0.05 (^ns^ non-significant).

## 3. Results

### 3.1. Clinical Relevance of Mortalin Upregulation in Head and Neck Cancer Patients

**Figure 1:**
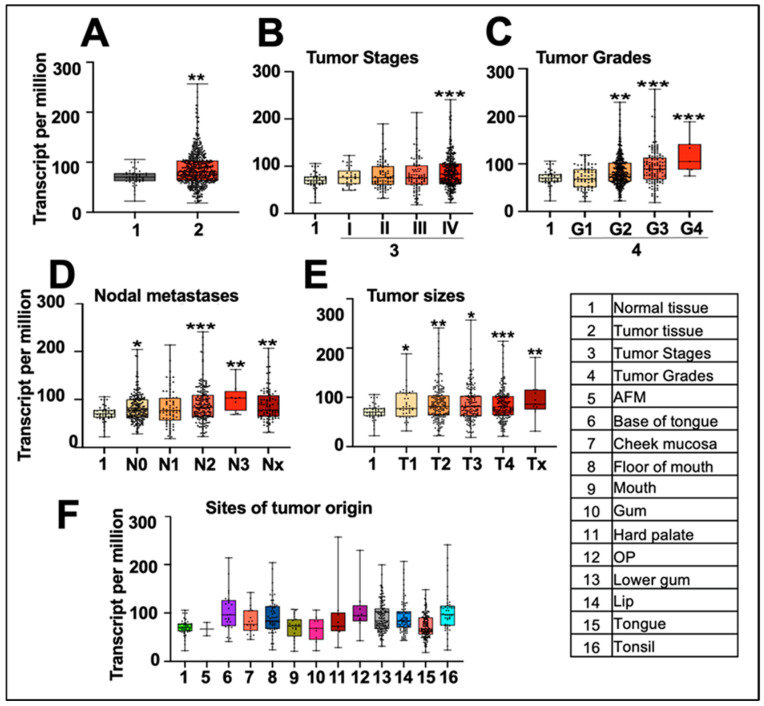
Mortalin is highly overexpressed in head and neck cancer—in silico analysis. (**A**–**F**) In silico analysis of mortalin expression in oral cancer tissue samples obtained from the TCGA dataset. (**A**) Comparison of mortalin expression in normal versus tumor samples. Expression in different categories of head and neck cancer, such as stages (**B**), grades (**C**), nodal metastases (**D**), and tumor sizes (**E**). (**F**) Expression at the various sites of tumor origin in the oral cavity. *p*-values < 0.001 (*** highly significant), <0.01 (** very significant), <0.05 (* significant).

To examine the clinical relevance of mortalin in head and neck cancer, we initially evaluated its mRNA expression levels in normal (*n* = 44) and tumor (*n* = 520) tissues from the TCGA database using R programming. The data (transcripts per million) shown in Figure 1A revealed significant upregulation in tumor tissues compared to normal tissues. Additionally, mortalin demonstrated notable upregulation in samples of stage IV, grades (G2, G3, and G4), lymph node metastasis, and larger tumor sizes (Figure 1B–E). As shown in Figure 1F, we found an increase in mortalin at different tumor sites. Tumors located at the base of the tongue (BT), cheek mucosa (CM), floor of the mouth (FM), hard palate (HP), overlapping lesions of the lip, oral cavity, pharynx (OP), lip, lower gum (LG), and tonsil (TN) exhibited higher levels of mortalin expression. In contrast, the anterior floor of the mouth (AFM) and tongue (TG) did not show any significant difference compared to normal tissue (Figure 1F).

**Figure 2:**
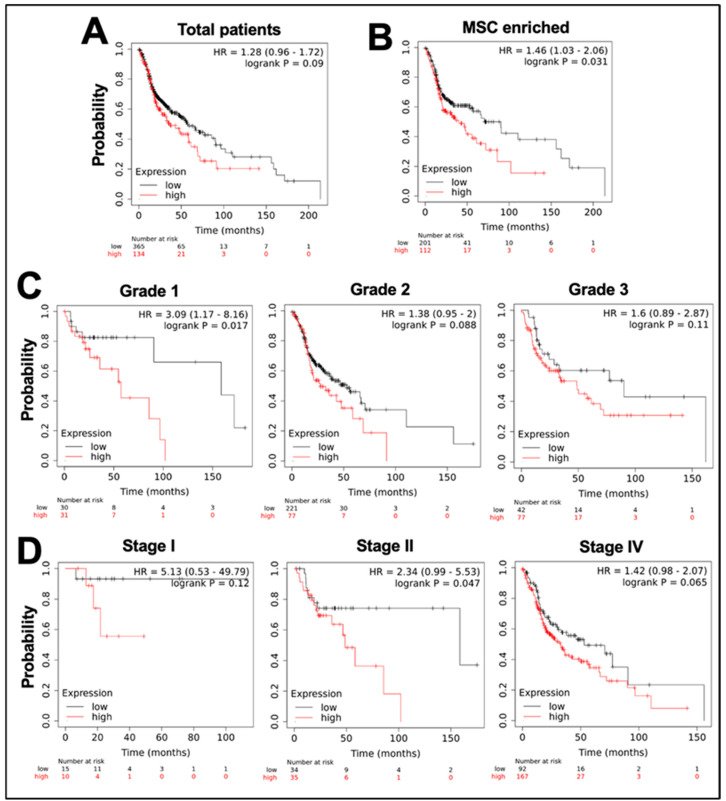
Mortalin overexpression correlates with poor survival in head and neck cancer patients. Overall survival (OS) analysis of oral cancer patients represented in Kaplan–Meier survival curves, demonstrating OS analysis of patients classified by mortalin expression levels (high vs. low) in various categories: (**A**) Total patients [HR = 1.28 (0.96–1.72)], (**B**) Patients with enriched mesenchymal stem cells (MSCs) in TME [HR = 1.46 (1.03–2.06)], (**C**) Tumor Grades 1, 2, and 3, [HR = 3.09 (1.17–8.16), HR = 1.38 (0.95–2), HR = 1.6 (0.89–2.87)] (**D**) Tumor Stages I, II, and IV, [HR = 5.13 (0.53–49.79), HR = 2.34 (0.99–5.53), HR = 1.42 (0.98–2.07)], respectively.

We next examined the significance of mortalin overexpression on the survival of head and neck cancer patients and found its tight correlation with poor survival of the patients. As shown in Figure 2A, overall survival (OS) analysis among patients with low and high levels of mortalin mRNA showed poorer survival in the latter group [HR 1.28 (0.96–1.72); *p*-value 0.09], demonstrating an inverse relationship between mortalin expression and patient survival. We also analyzed OS in patients with high versus low mortalin expression in tissues enriched with mesenchymal stem cell (MSC) markers. We found significantly lower OS in patients with high mortalin levels as compared to those having low mortalin expression (*p* = 0.031) (Figure 2B). A similar trend of low survival was observed in patients with higher levels of mortalin in grade 1 (*p*-value = 0.017), grade 2 (*p*-value = 0.088), and grade 3 (*p*-value = 0.11) (Figure 2C), stage I (*p*-value = 0.12), stage II (*p*-value = 0.042), and stage IV (*p*-value = 0.065) (Figure 2D) of the disease.

**Figure 3:**
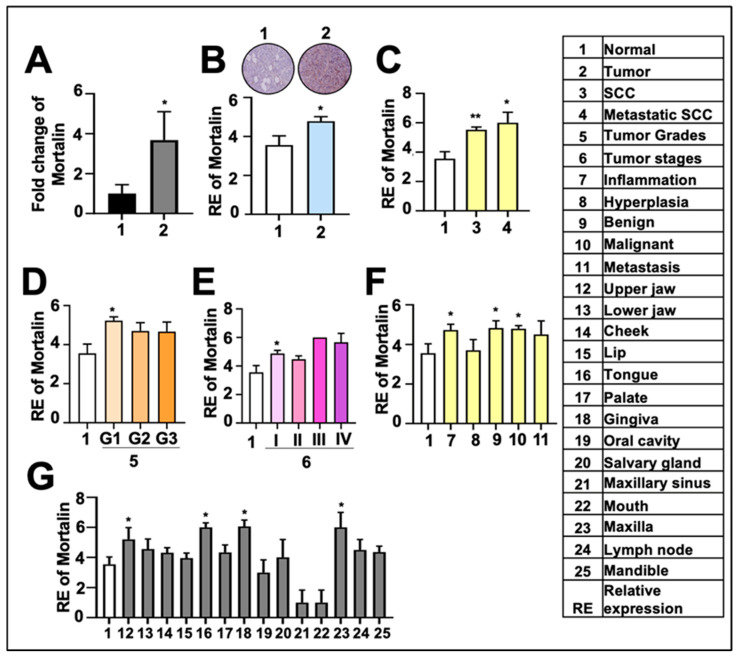
Mortalin is highly overexpressed in oral cancer, as shown in in vitro analysis. (**A**) Fold change of mortalin in oral cancer patient samples obtained from Northeast India. (**B**–**G**) IHC analysis revealing mortalin expression in oral cancer tissue samples. (**B**) Representative images and relative expression (RE) of mortalin in normal vs tumor samples. RE of mortalin in squamous cell carcinoma (SCC) and metastatic SCC tissue samples (**C**); different grades (**D**); different stages of oral cancer (**E**); tissue samples categorized by different pathological stages of oral carcinogenesis (**F**); different tissues of tumor origin (**G**). *p*-values < 0.01 (** very significant), <0.05 (* significant).

To support the above TCGA results, we next determined the mortalin mRNA expression in oral cancer tissue samples obtained from the Northeast region of India. As shown in Figure 3A, tumor tissue (*n* = 21) samples showed 3–4-fold higher levels of mortalin mRNA as compared to the normal tissue (*n* = 4) samples. To further validate the frequent upregulation of mortalin in oral cancers, we used a tissue microarray slide containing normal oral and neoplasm tissue sections (details in Table S1). Consistent with the in-silico findings, IHC of the microarray samples showed upregulation of mortalin in oral tumor tissues compared to the normal tissues (Figure 3B). The overexpression of mortalin was also observed in SCC and metastatic SCC tissues (Figure 3C). Moreover, IHC analysis demonstrated overexpression of mortalin across grade I (Figure 3D) and stage I (Figure 3E), as well as different pathological conditions of oral carcinogenesis, such as inflammation and benign and malignant tumors; however, the expression was not significant in hyperplasia and metastatic tumors included in this study (Figure 3F). We also determined mortalin expression in tumors with different anatomic origins (Figure 3G). We found its upregulation in most regions, including the upper and lower jaw, cheek, lip, tongue, palate, gingiva, salivary gland, maxilla, and lymph node. These data predicted an important role of mortalin in oral cancer survival and progression to aggressive stages.

### 3.2. Knockdown of Mortalin Caused Apoptosis in Oral Cancer Cells

**Figure 4:**
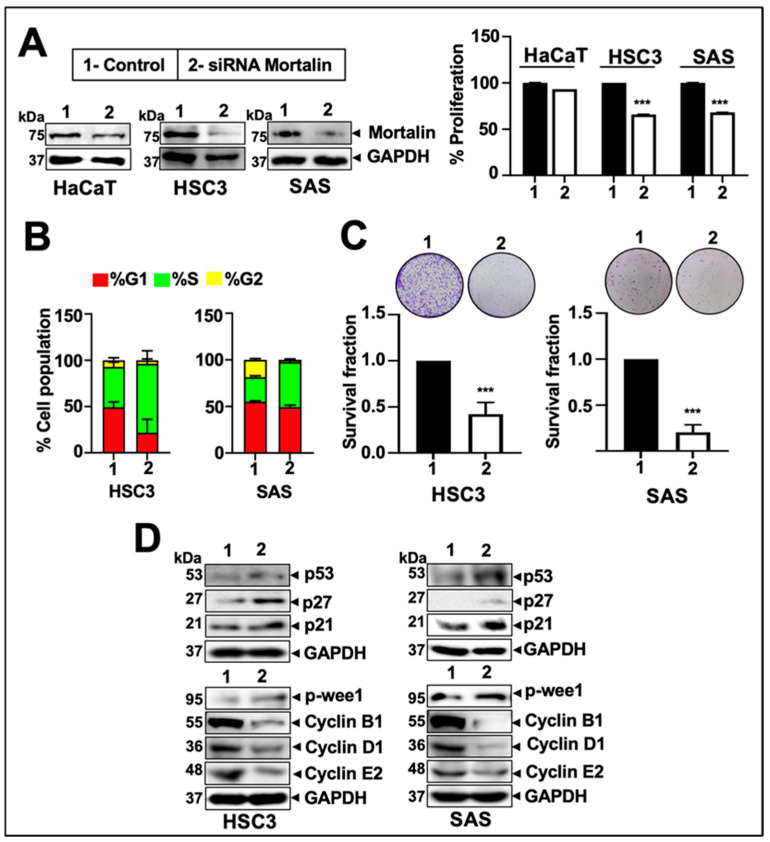
Knockdown of mortalin inhibited cell survival and proliferation in oral cancer. (**A**) Represents the successful knockdown of mortalin through siRNA in HaCaT, SAS, and HSC3 cell lines, followed by the bar graph showing percentage inhibition in cell proliferation in HaCaT, HSC3, and SAS cells. (**B**) Demonstrate the impact of mortalin knockdown on the cell cycle in HSC3 and SAS cells. (**C**) Images depict the colonies (blue/purple dots) formed in HSC3 and SAS cells, represented in bar graphs showing the survival fraction. (**D**) Expression of proteins associated with cell survival and proliferation was assessed via immunoblotting in knockdown samples. *p*-values < 0.001 (*** highly significant).

To investigate the molecular mechanism and therapeutic efficacy of mortalin targeting in oral cancer, we examined its expression in HSC3 and SAS oral cancer cells compared to normal keratinocytes, HaCaT (Figure S1, Document S1). As shown in Figure S1A–C, both the mortalin protein and mRNA were upregulated in oral cancer compared to normal cells. Next, we performed siRNA-mediated silencing of mortalin in both oral cancer and normal cells (Figure 4A and Figure S3). While siRNA-mediated knockdown of mortalin in HSC3 and SAS cells resulted in a significant decrease in their proliferation, confirmed by MTT assay, HaCaT cells showed only a 5–10% decrease (Figure 4A).

Mortalin knockdown with different concentrations of siRNA caused a dose-dependent increase in apoptosis, as evidenced by DAPI and PI staining (Figure S2). Since the cell cycle plays a crucial role in cancer cell proliferation, cell cycle analysis was conducted to investigate the role of mortalin in regulating various cycle events. The cell cycle analysis of control and mortalin-knockdown cells revealed the induction of cell cycle arrest at the S-phase in both HSC3 and SAS cells (Figure 4B), indicating disrupted progression during the DNA synthesis phase. These data suggest that mortalin is essential for completing the S-phase and plays a critical role in supporting the proliferation of oral cancer cells. Furthermore, siRNA-mediated knockdown of mortalin inhibited the clonogenic potential of both HSC3 and SAS oral cancer cells (Figure 4C). The number of colonies formed in the mortalin-knockdown cells was decreased compared to the control, demonstrating a significant decrease in their survival and proliferation, key criteria for tumorigenicity and progression to aggressive stages. 

**Figure 5:**
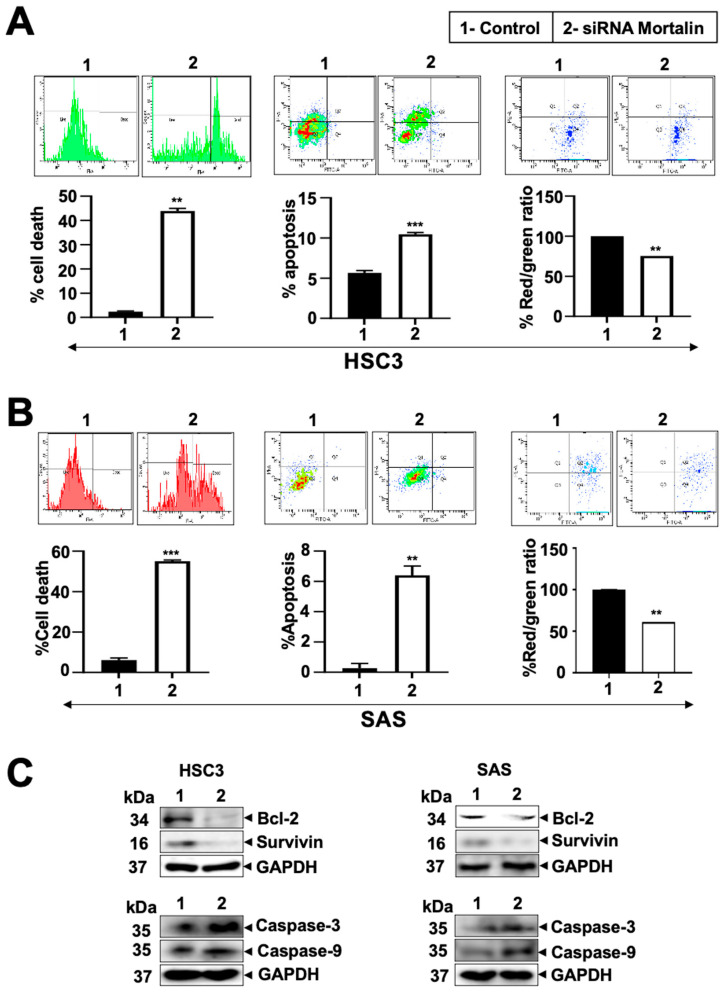
Knockdown of mortalin induced cell death in oral cancer. Induction of cell death, apoptosis, and decrease in mitochondrial potential (represented by the red/green ratio) in control and siRNA-treated HSC3 (**A**) and SAS (**B**) cells. (**C**) Expression of proteins involved in regulating apoptosis. *p*-values < 0.001 (*** highly significant), <0.01 (** very significant).

Western blot analysis for proteins involved in cell cycle progression revealed increased levels of p53, p27, p21WAF1, and p-wee1 in cells deficient in mortalin expression. Notably, these cells showed a decrease in the expression of cyclin B1, cyclin D1, and cyclin E2 compared to the control. Similar data were obtained in both the oral cancer cell lines (Figure 4D and Figure S4). These data established that the essential function of mortalin is the sustained proliferation of oral cancer cells. To decipher the mechanism of mortalin knockdown-induced decrease in cell proliferation, we next performed PI-FACS and annexin V apoptosis assays in control and mortalin-compromised cells (at ~25 pM siRNA). As represented in Figure 5A,B (left and middle panels in each figure), mortalin-compromised cells underwent apoptosis, as also supported by Western blot analysis (Figure 5C and Figure S5). Mortalin-compromised cells exhibited a significant reduction in the antiapoptotic proteins Bcl-2 and Survivin, as well as an increase in markers of apoptosis, including cleaved Caspase-3 and Caspase-9. Additionally, a JC-1 assay was performed to determine the state of mitochondrial health in both oral cancer cells, which showed a decrease in the ratio of JC-1 aggregates to monomers (Figure 5A,B; right panel), indicating loss of mitochondrial membrane potential in knockdown cells. 

### 3.3. Knockdown of Mortalin Attenuated EMT, Angiogenesis, Invasion, and Migration in Oral Cancer Cells

**Figure 6:**
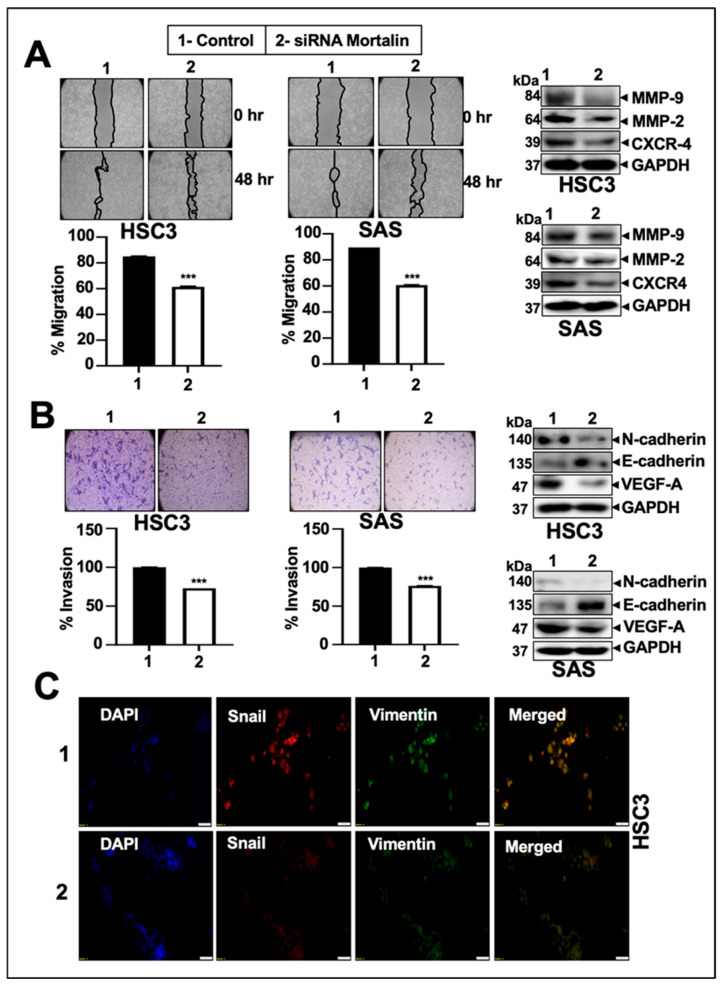
Knockdown of mortalin suppressed EMT, angiogenesis, migration, and invasion in oral cancer. The figure represents the migration (**A**) and invasion (**B**) in HSC3 and SAS mortalin knockdown cells. The immunoblotting images show the expression of proteins associated with these hallmarks, including EMT and angiogenesis (**A**,**B**, right panel). (**C**) Immunocytochemistry (ICC) assay determining the expression/intensity of EMT markers in HSC3 cells through fluorescence imaging. *p*-values < 0.001 (*** highly significant).

We next determined the effect of the mortalin silencing on the migration and invasion characteristics of oral cancer cells. Cells were treated with nontoxic concentrations of mortalin siRNA that did not cause apoptosis as determined by independent experiments (Figure S2). Lack of cytotoxicity was also confirmed by microscopic observations during the migration and invasion assays. Following 48 h of transfection, the cells were maintained and reseeded in serum-free conditions for the experiments. Wound scratch assays showed a decrease in migration capacity of mortalin-compromised cells as compared to the control cells (Figure 6A). The Boyden chamber assay revealed that silencing of mortalin significantly suppressed the invasion of oral cancer cells (Figure 6B). These phenotypic changes were well supported by molecular data, including the downregulation of MMP-9, MMP-2, CXCR-4, N-cadherin, and VEGF-A. E-cadherin showed an increased expression (Figure 6A,B, right panel, and Figure S6). Snail and Vimentin, which are involved in dedifferentiation, showed a remarkable reduction (Figure 6C).

### 3.4. Knockdown of Mortalin Modulated Akt/mTOR Signaling Cascades in Oral Cancer Cells

Akt/mTOR has been established as a key molecular marker and therapeutic target of oral cancer. Hence, we investigated the role of mortalin in this signaling pathway. 

**Figure 7:**
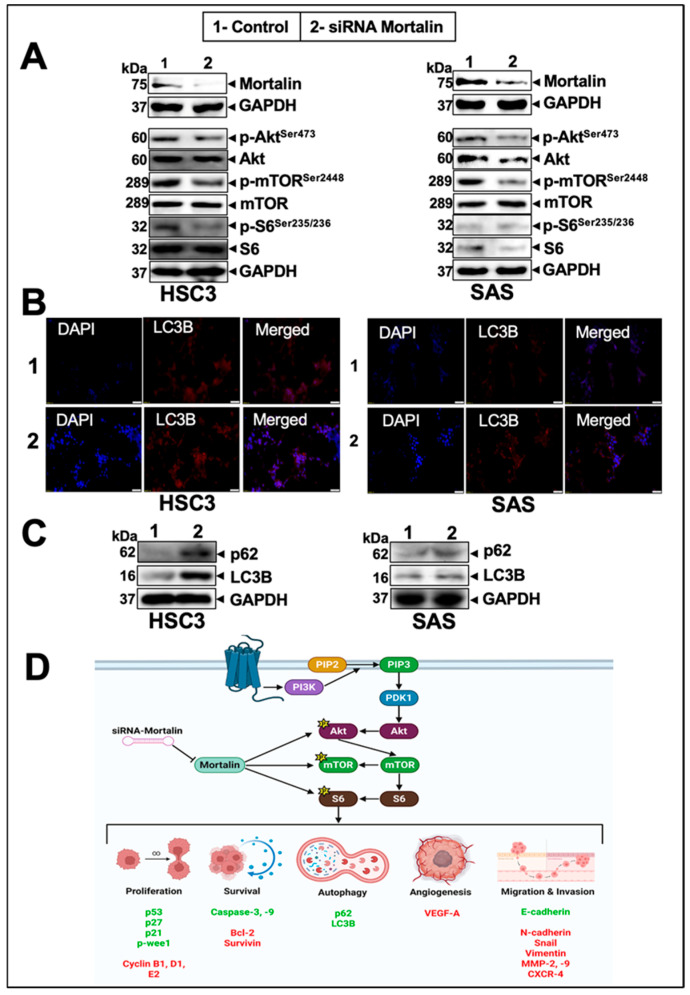
Knockdown of mortalin modulated Akt signaling and autophagic proteins in oral cancer. (**A**) Expression of Akt signaling proteins in HSC3 and SAS cells, in control vs. siRNA-treated samples. (**B**) The ICC assay is used to determine the expression of the autophagic marker (LC3B) through fluorescence imaging in control and siRNA-treated samples for HSC3 and SAS cells. (**C**) Expression of proteins associated with autophagy, upon the knockdown of mortalin in HSC3 and SAS cells. (**D**) Schematic representation of the putative mechanism of mortalin in oral cancer. Upregulated hallmark proteins upon knockdown are shown in green, while suppressed proteins are shown in red.

Western blotting of control and mortalin-compromised HSC3 and SAS cells showed a remarkable reduction in the expression of *p*-Akt^Ser473^ and *p*-mTOR^Ser2448^ compared to control (Figure 7A and  Figure S7). Notably, *p*-S6^Ser235/236^ expression was also downregulated in mortalin-knockdown cells, suggesting that mortalin acts as an upstream regulator of Akt/mTOR signaling, which promotes proliferation, migration, EMT, invasion, and angiogenesis, while inhibiting autophagy in cancer cells. Consistent with these, mortalin-compromised cells showed an increase in p62 and LC3B, signifying modulation of autophagy (Figure 7B,C and Figure S7). Taken together, the given data suggest a key role of mortalin in the Akt/mTOR-driven continued proliferation of oral cancer cells by inhibiting apoptosis and autophagy (Figure 7D). These data strongly suggested mortalin as a prognostic biomarker and a therapeutic target for oral cancer. 

## 4. Discussion

Overexpression of mortalin in cancer cells has been shown to modulate their major characteristics, including unrestricted proliferation, survival, enhanced EMT, invasion, angiogenesis, migration properties leading to metastasis, and drug resistance [12,14,26,40]. High expression of mortalin has earlier been shown to be consistently associated with poor prognosis and survival in malignancies such as breast, brain, cholangiocarcinoma, colon, gastric, hepatocellular, lung, pancreatic, prostate, and thyroid cancers [14,24,25,28,29,30,31,32,33]. However, its involvement in oral cancers remains unexplored. This study is the first one, to the best of our knowledge, to report a significant correlation of mortalin upregulation with oral cancer progression and poor prognosis. Of note, higher levels of mortalin expression were detected across various subgroups of patients, characterized by stage, grade, tumor size, lymph node metastasis, and other pathological conditions of oral carcinogenesis, except hyperplasia. Moreover, mortalin expression was particularly higher in specific anatomical sites such as the upper jaw, tongue, gingiva, and maxilla. These data endorsed that mortalin enrichment was clinically relevant and correlated well with the progression of oral cancer to advanced stages as defined by histological grades, clinical stages, and lymph node metastasis. Furthermore, analysis using TCGA data for oral cancer patients revealed that mortalin overexpression was associated with poor OS of the oral cancer patients, particularly in patients with enriched mesenchymal stem cell markers. Based on this and earlier reports showing the effect of mortalin overexpression on EMT signaling and cancer cell stemness [26], we examined the status of molecular markers of EMT in oral cancer cells compromised for mortalin expression. Consistent with the reduced ability of mortalin-knockdown cells to proliferate, migrate, and invade, as observed by phenotypic assays, we found S-phase cell cycle arrest and a significant decline in clonogenic survival, supporting attenuation of long-term proliferation. Notably, similar antiproliferative effects have been reported with agents such as 5-FU and Vorinostat in inducing S-phase arrest in oral cancer, reinforcing the importance of targeting S-phase regulatory mechanisms in cancer therapy [41,42]. In our study, these effects were mediated through the activation of tumor suppressor p53, CDK inhibitors, and checkpoint proteins (such as *p*-wee1, p27, p21WAF1, and p18) as well as caspases and the epithelial marker E-cadherin. Conversely, there was a suppression of cyclins (B1, D1, and E2), Bcl-2, Survivin, MMPs (-2 and -9), N-cadherin, CXCR-4, Snail, Vimentin, and VEGF-A. Importantly, mortalin knockdown did not significantly affect the proliferation of normal HaCaT cells, suggesting that mortalin is a cancer cell-specific target, which could be attributed to the cancer cell-specific mortalin-p53 interaction reported earlier [27].

Activation of the Akt/mTOR pathway has been demonstrated as a major pathway involved in oral cancer, its lymph node metastasis, progression to advanced stages, chemoresistance, radioresistance, and poor survival outcomes [9,37,38,43,44,45,46]. This pathway is also upregulated by agents like tobacco and its components, benzo(a)pyrene, and nicotine [37,38], the major cause of the high rate of oral cancer in the Northeast region of India. Interestingly, various plant extracts, natural compounds, and inhibitors, such as guggulsterone, triphala, LY294002, and oridonin, have been shown to function as inhibitors of Akt/mTOR signaling, yielding cell cycle arrest and/or apoptosis [47,48,49,50]. BEZ235, an inhibitor of PI3K/Akt/mTOR signaling and the cyclin D1/CDK-4 complex, was shown to cause synergistic radiosensitization in oral cancer cells [46]. Mortalin was shown to regulate drug resistance pathways mediated by activated Akt, HIF-1α, and c-Myc proteins [51,52]. Consistently, knockdown of mortalin was shown to result in sensitization of cancer cells to cisplatin [51]. Furthermore, mortalin inhibitors, including peptides, RNAs, and synthetic and natural small molecules (such as MKT-077, CAPE, embelin, salvianolic acid-B, mortaparib, mortaparib^Plus^, mortaparib^Mild^, fucoxanthin, UBXN2A, Az-TPP-03, and SHetA2), have shown significant anti-cancer activity mediated by disruption of the mortalin-p53 complex, causing reactivation of p53 function [33,53,54,55,56,57,58,59,60,61,62,63,64,65]. Their effect on Akt/mTOR signaling, a key driver of oral carcinogenesis, has not been fully defined and hence warrants comprehensive laboratory and clinical attention, leveraging new treatment modalities for oral cancers.

Despite the strengths of this study, a few limitations should be acknowledged. Although this study investigated mortalin expression in oral cancer tissues from the Northeast region of India, a more comprehensive analysis involving samples from diverse geographical regions worldwide would strengthen the findings. Furthermore, assessing mortalin expression across a larger cohort and various anatomical sub-sites within the oral cavity could offer more comprehensive insights. Studies could also explore its correlation with different mutational markers such as TP53, PTEN, and HRAS. However, it is also noteworthy to mention that the consistent knockdown efficiency and reproducible phenotypic outcomes across multiple assays support the specificity of the observed effects. Although our in vitro experiments demonstrated that mortalin silencing inhibited key hallmarks of oral cancer, such as cell survival, proliferation, EMT, angiogenesis, migration, and invasion, these findings were based on two oral cancer cell lines. Studies with a broader panel of cell lines, and importantly, validating the potential of mortalin as a therapeutic target through in vivo models, are warranted. These efforts will be essential for translating the findings toward clinical application.

## 5. Conclusions

In silico and experimental data demonstrated that mortalin upregulation in oral cancers is clinically relevant. Mortalin plays a significant role in activating survival, proliferation, EMT, migration, invasion, and angiogenesis signaling, as well as poor overall survival of cancer patients. Mortalin siRNA could reverse these pathways, potentially through the inhibition of Akt/mTOR signaling, a key oncogenic pathway that activates oral carcinogenesis. The study proposes mortalin as a promising therapeutic target for oral cancers and urges the need for the development of mortalin inhibitors and their validation in laboratory and clinical settings.

## Data Availability

All study data are provided within the article and the Supplementary Information. In silico analyses were conducted using publicly accessible datasets from The Cancer Genome Atlas.

## Supplementary Materials

The following supporting information can be downloaded at: https://www.mdpi.com/article/10.3390/cancers17172860/s1, Figure S1: Expression of mortalin in oral cancer cell lines; Figure S2: (A) Western blot images for mortalin expression with different concentrations of siRNA. (B & C) DAPI and PI staining of oral cancer cells with increasing doses of mortalin-specific siRNA. Higher concentrations resulted in increased cell death, as indicated by PI (red) staining; Figure S3: Supplementary to Figure 4A; Figure S4: Supplementary to Figure 4D; Figure S5: Supplementary to Figure 5C; Figure S6: Supplementary to Figure 6A,B; Figure S7: Supplementary to Figure 7; Table S1: Patient specification of Oral Cancer Tissue Microarray samples; Table S2: Scoring method for IHC; Table S3: Details of head and neck cancer patient samples collected from Northeast India; Table S4: Details of primary and secondary antibodies; Document S1: Original Western blots.
