# Supplementary material for: Mortalin Represents a Promising Therapeutic Target for Oral Cancers: Clinical Relevance and Experimental Evidence for the Activation of Akt/mTOR Signaling
Source: Cancers (Basel). 2025 Aug 30;17(17):2860. doi: 10.3390/cancers17172860 (PMC12427376; doi:10.3390/cancers17172860)
Supplement: Supplementary file 1 [file cancers-17-02860-s001.zip › cancers-3765961-Document S1.pdf]

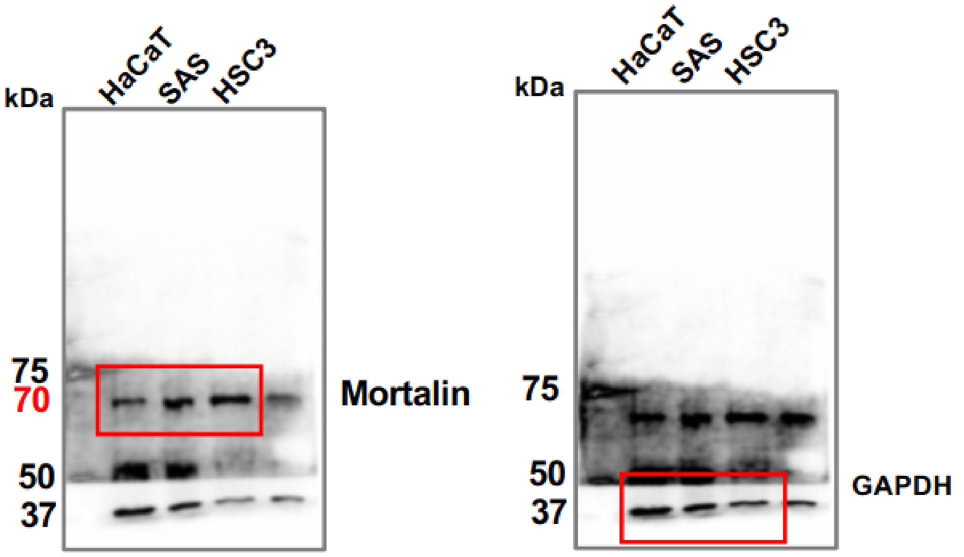

Full Western blots for the protein of interest (Mortalin) in normal (HaCaT) and oral cancer cells- SAS and HSC3 (Supplementary Figure S1A). GAPDH (36-kDa) was used as a loading control.

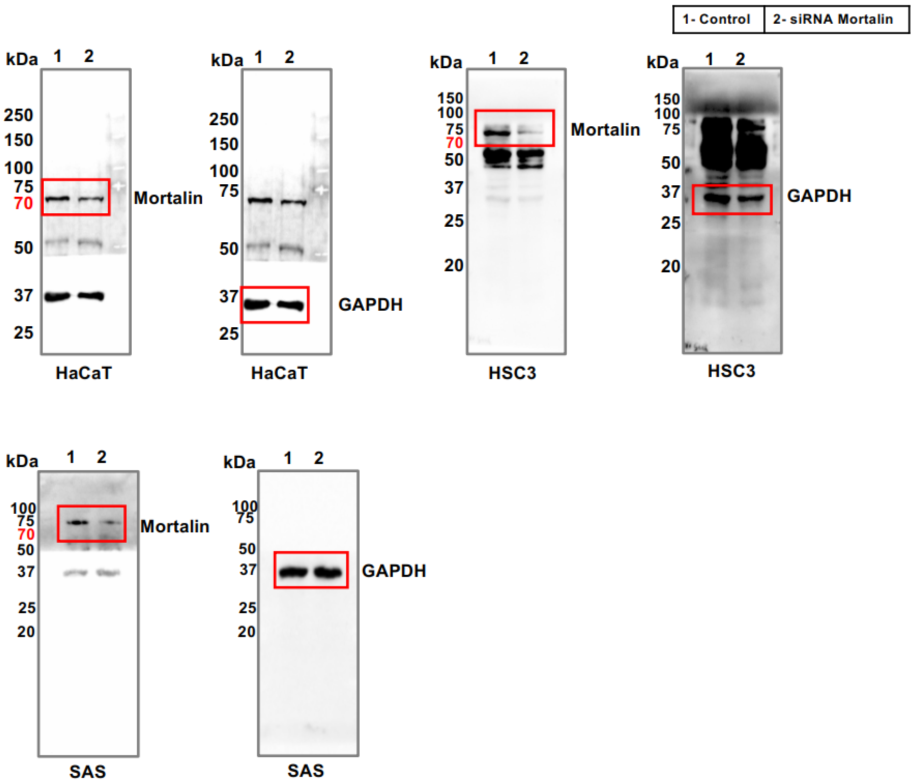

Full Western blots for the protein of interest (Mortalin) in normal (HaCaT) and oral cancer cells- HSC3 and SAS, treated with Mortalin - specific siRNA (Figure 4A). GAPDH (36- kDa) was used as a loading control

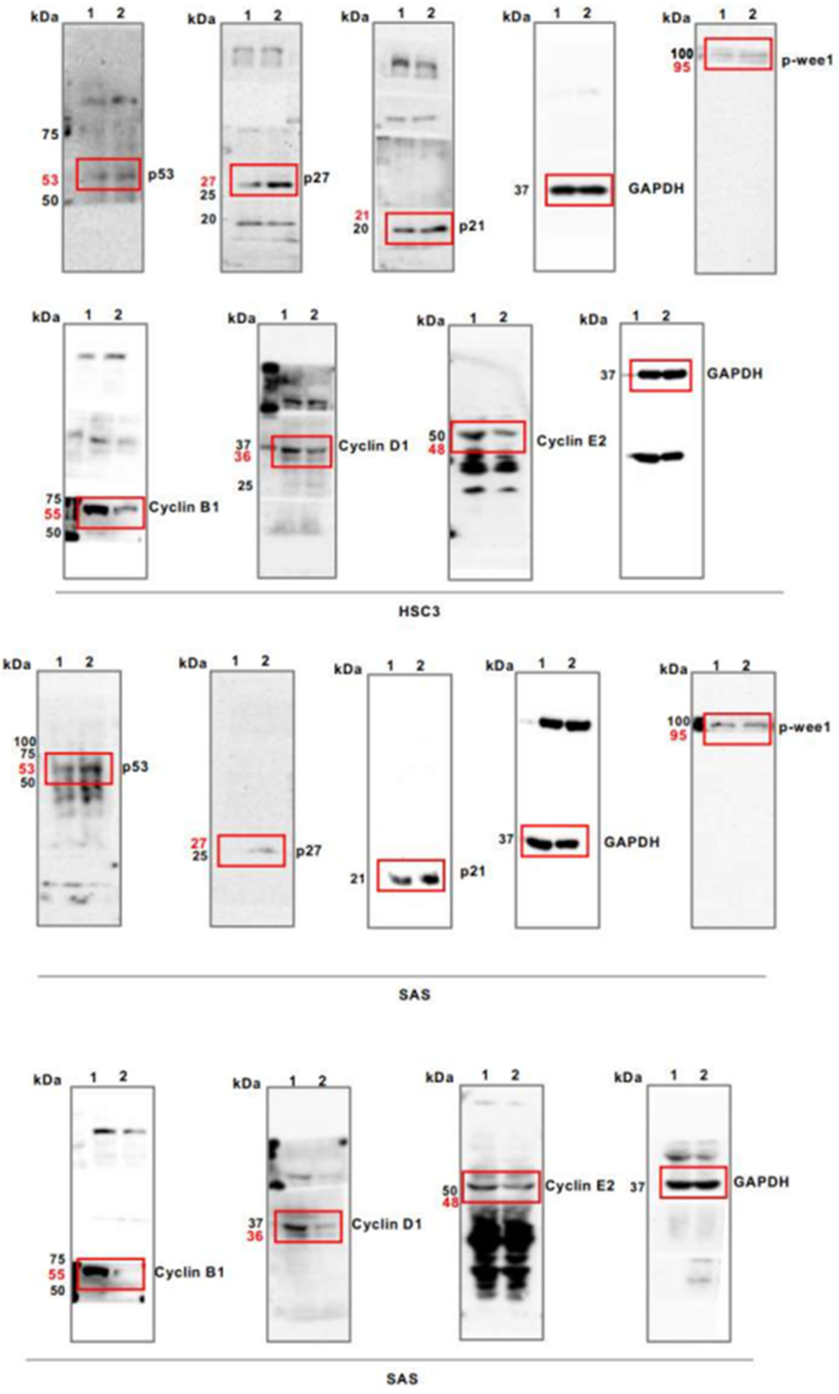

Full Western blots for the protein of interest (p53, p27, p21, p-wee1, Cyclin B1, Cyclin D1, Cyclin E2) in HSC3 and SAS cells treated with Mortalin specific siRNA (Figure 4D). GAPDH (36-kDa) was used as a loading control.

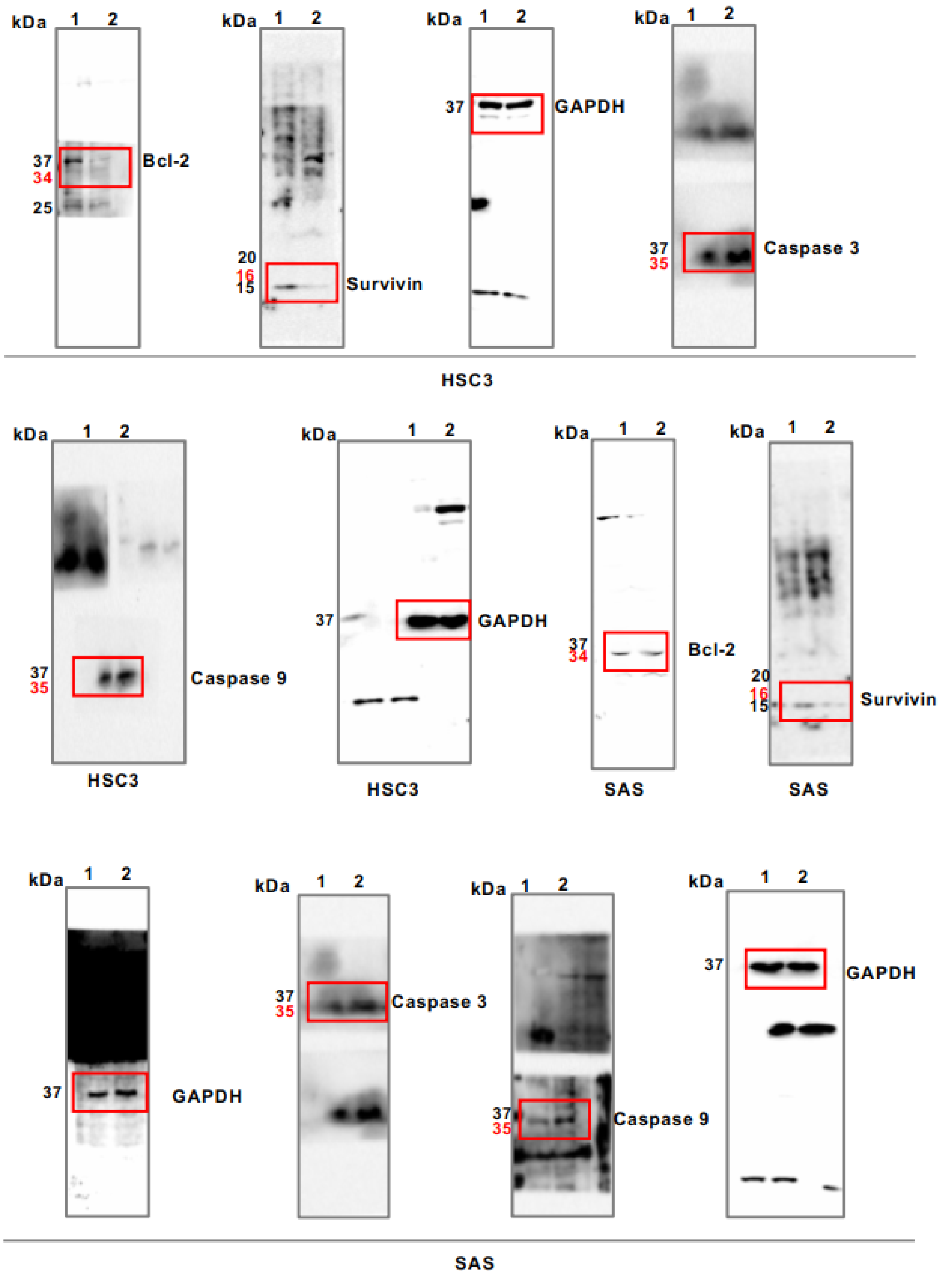

Full Western blots for the protein of interest (Bcl-2, Survivin, Caspase 3, Caspase 9) in HSC3 and SAS cells treated with Mortalin specific siRNA (Figure 5C). GAPDH (36-kDa) was used as a loading control

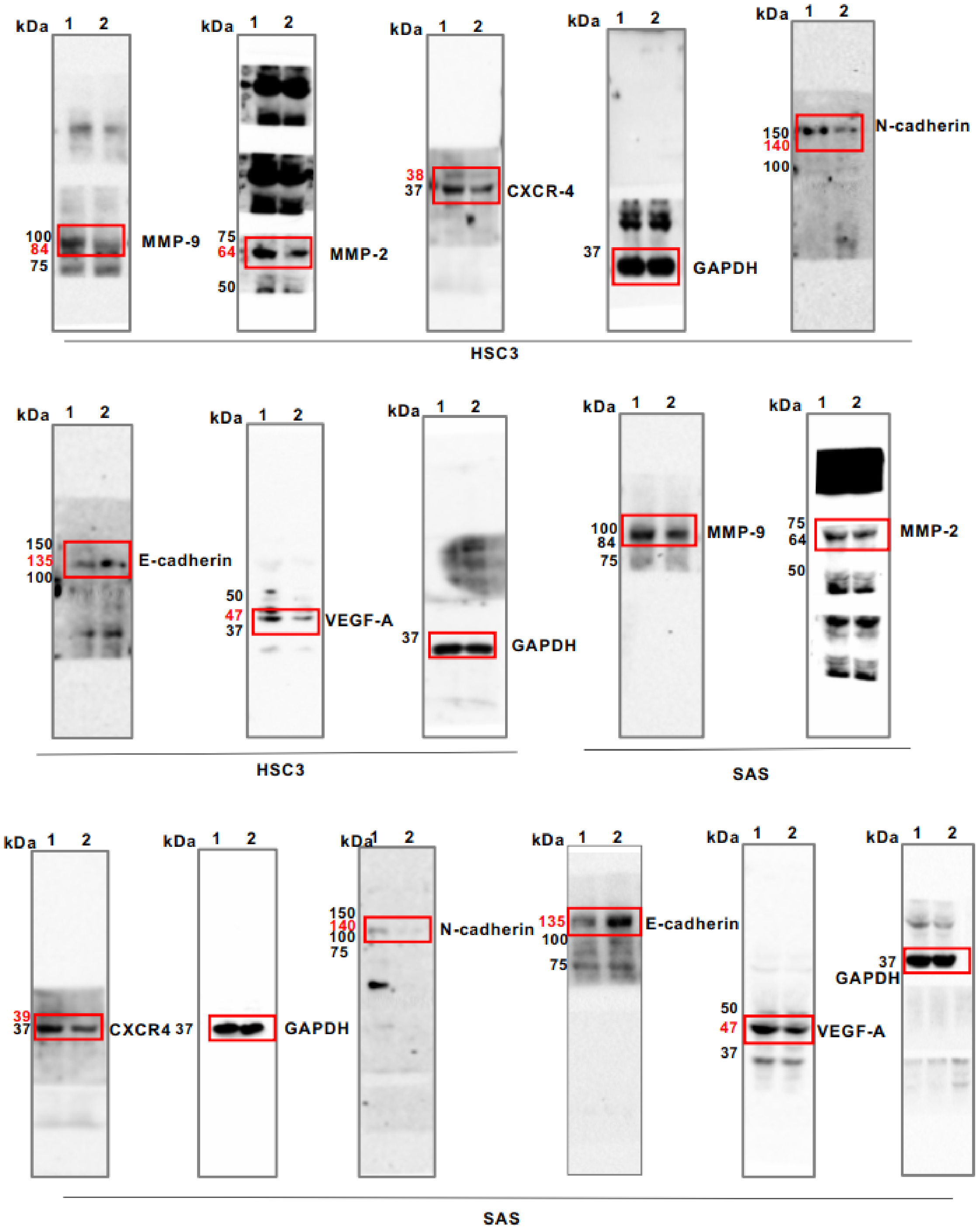

Full Western blots for the protein of interest (MMP-9, MMP-2, CXCR-4, Ncadherin, E-cadherin, VEGF-A) in HSC3 and SAS cells treated with Mortalin specific siRNA (Figure 6). GAPDH (36-kDa) was used as a loading control.

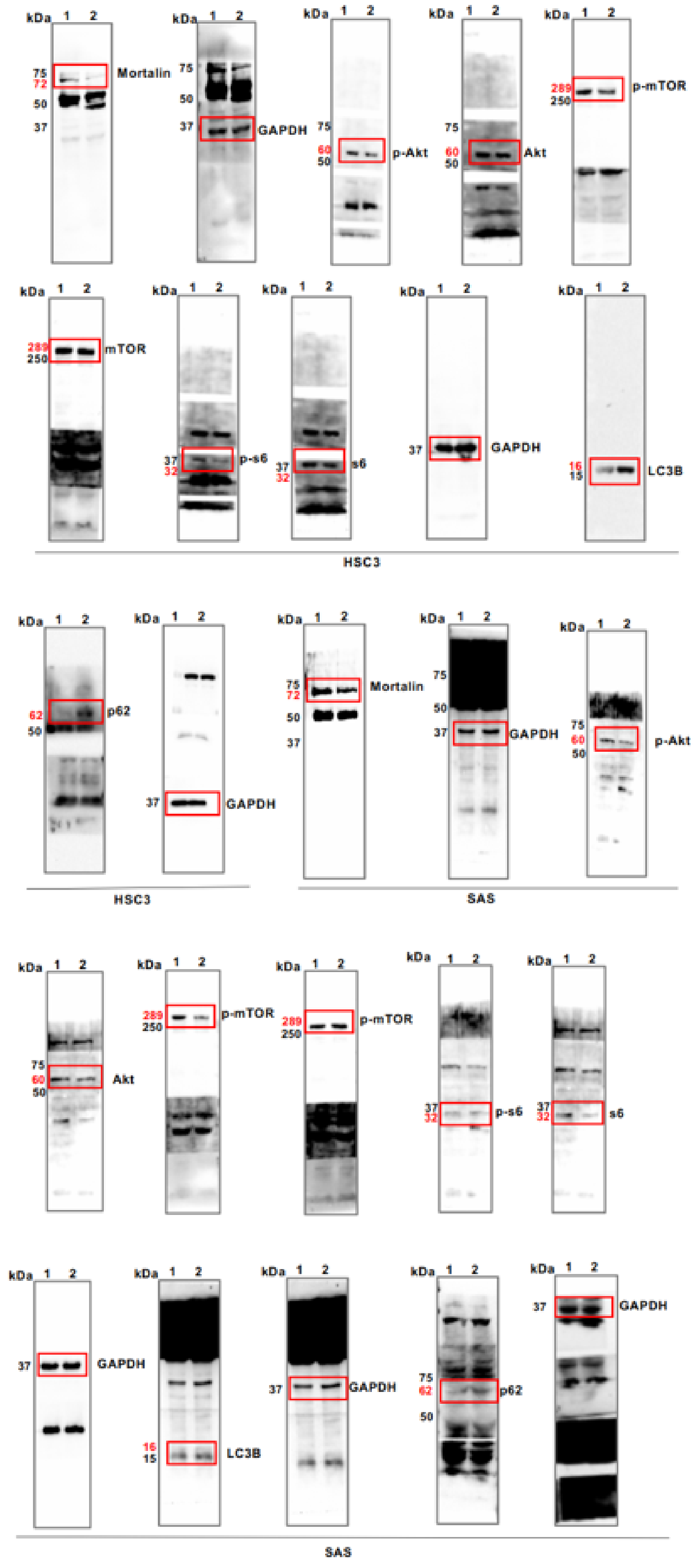

Full Western blots for the protein of interest (Mortalin, p-Akt, p-mTOR, ps6) in HSC3 and SAS cells treated with Mortalin specific siRNA (Figure 7A and 7C). GAPDH (36 - kDa) was used as a loading control.

*Only one GAPDH is represented for autophagic marker (LC3B, p62) for SAS cells in main Figure 7C
