# Supplementary material for: Mortalin Represents a Promising Therapeutic Target for Oral Cancers: Clinical Relevance and Experimental Evidence for the Activation of Akt/mTOR Signaling
Source: Cancers (Basel). 2025 Aug 30;17(17):2860. doi: 10.3390/cancers17172860 (PMC12427376; doi:10.3390/cancers17172860)
Supplement: Supplementary file 1 [file cancers-17-02860-s001.zip › cancers-3765961-supplementary Figures S1-S7.pdf]

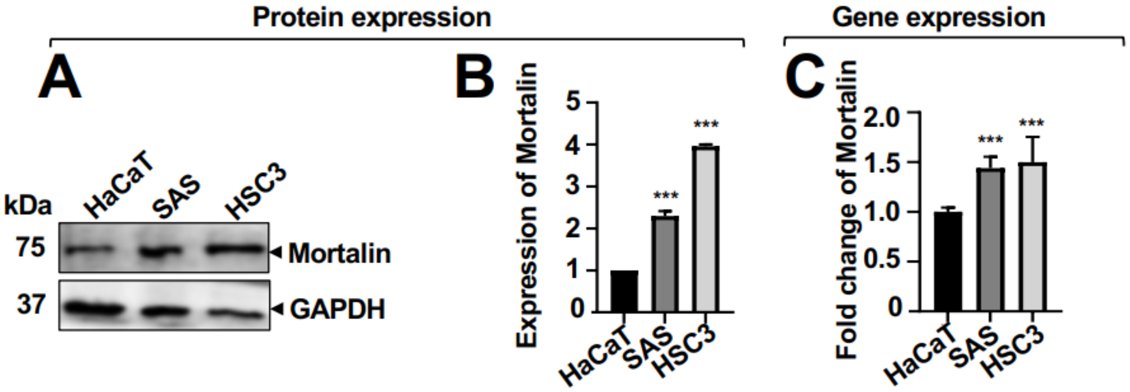

Figure S1. Expression of mortalin in oral cancer cell lines. (**A**) Immunoblotting images in normal vs oral cancer cell lines, determining the expression of mortalin. (**B**) Quantitative representative of supplementary figure 1A. (**C**) Fold change of mortalin mRNA in normal vs oral cancer cell lines. HaCaT cell line was used as a normal against SAS and HSC3 oral cancer cells. p-value < 0.001 (***highly significant).

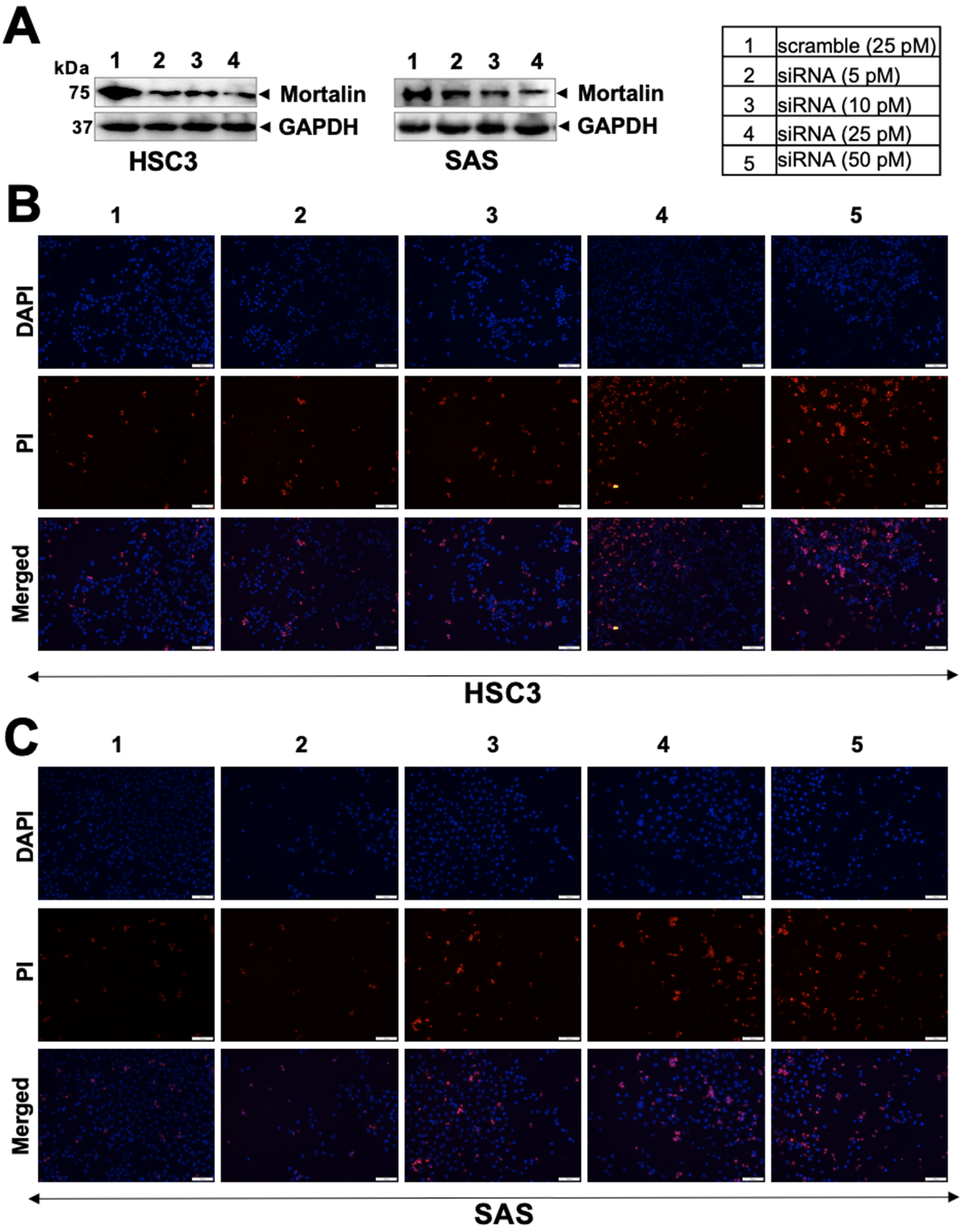

Figure S2: (A) Western blot images for mortalin expression with different concentrations of siRNA. (B & C) DAPI and PI staining of oral cancer cells with increasing doses of mortalin-specific siRNA. Higher concentrations resulted in increased cell death, as indicated by PI (red) staining.

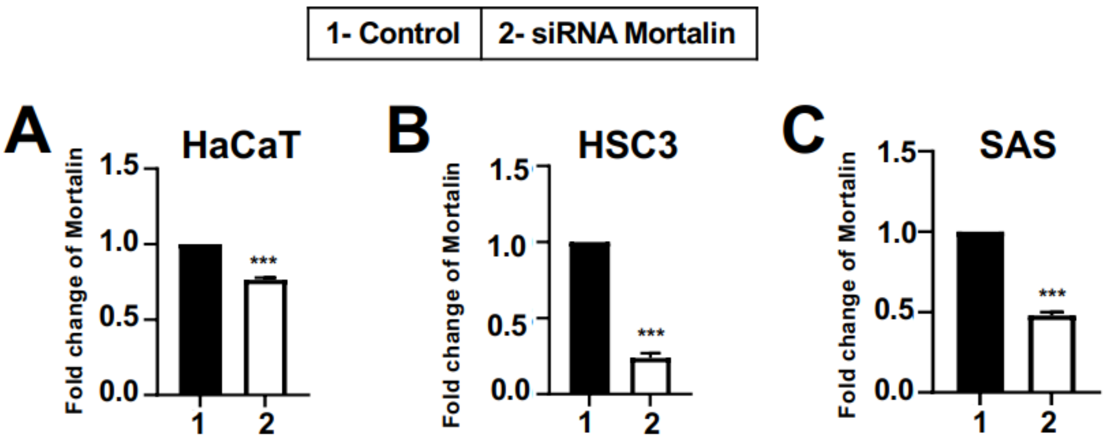

Figure S3 Supplementary to Figure 4A: Quantitative representation of the expression of mortalin in HaCaT, HSC3, and SAS cells subjected to siRNA-mediated knockdown of mortalin. *p*-value < 0.001 (***highly significant).

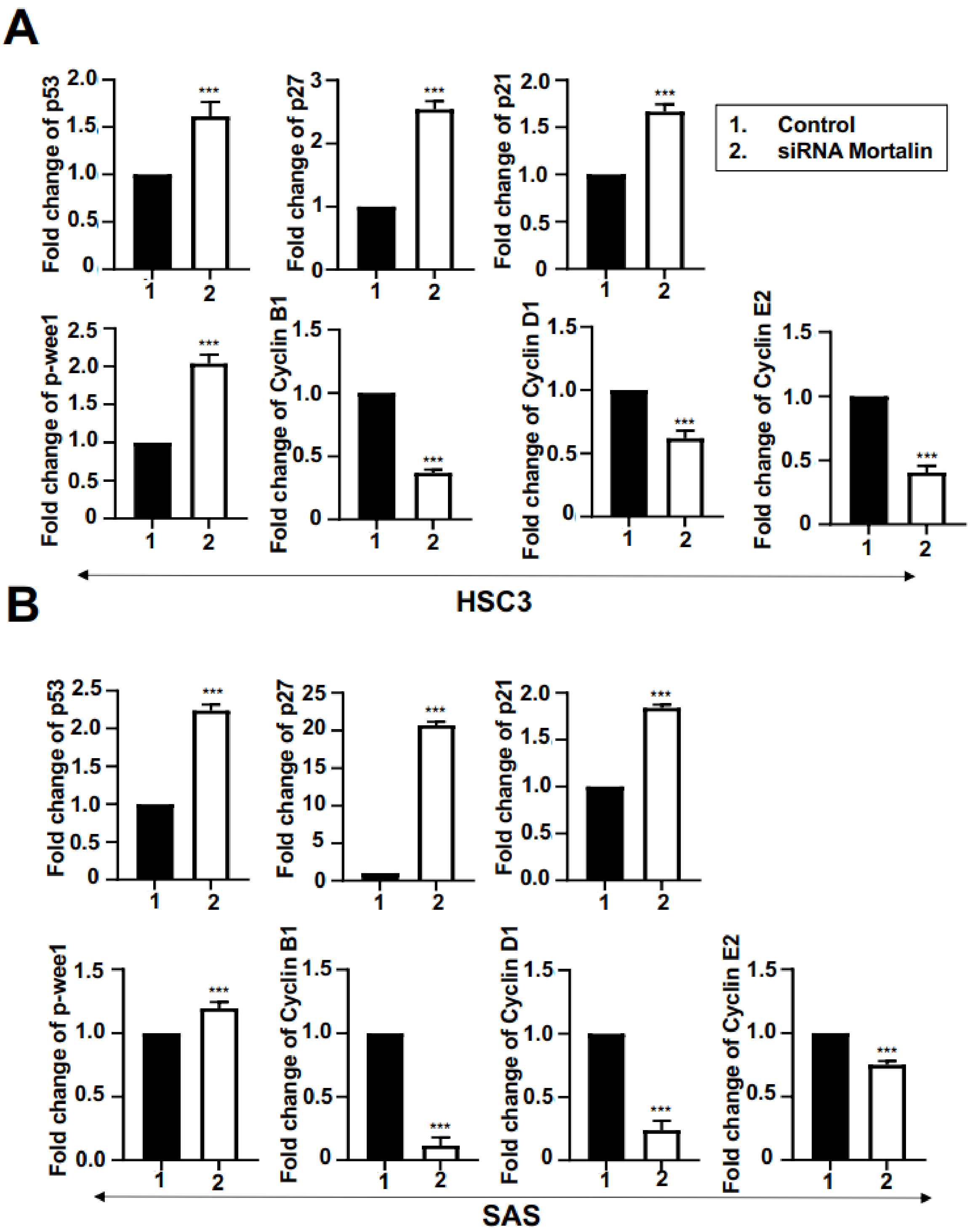

Figure S4. Supplementary to Figure 4D: Quantitative representation of the expression of proteins associated with cell survival and proliferation in HSC3 and SAS cells upon knockdown of mortalin. p-value < 0.001 (***highly significant).

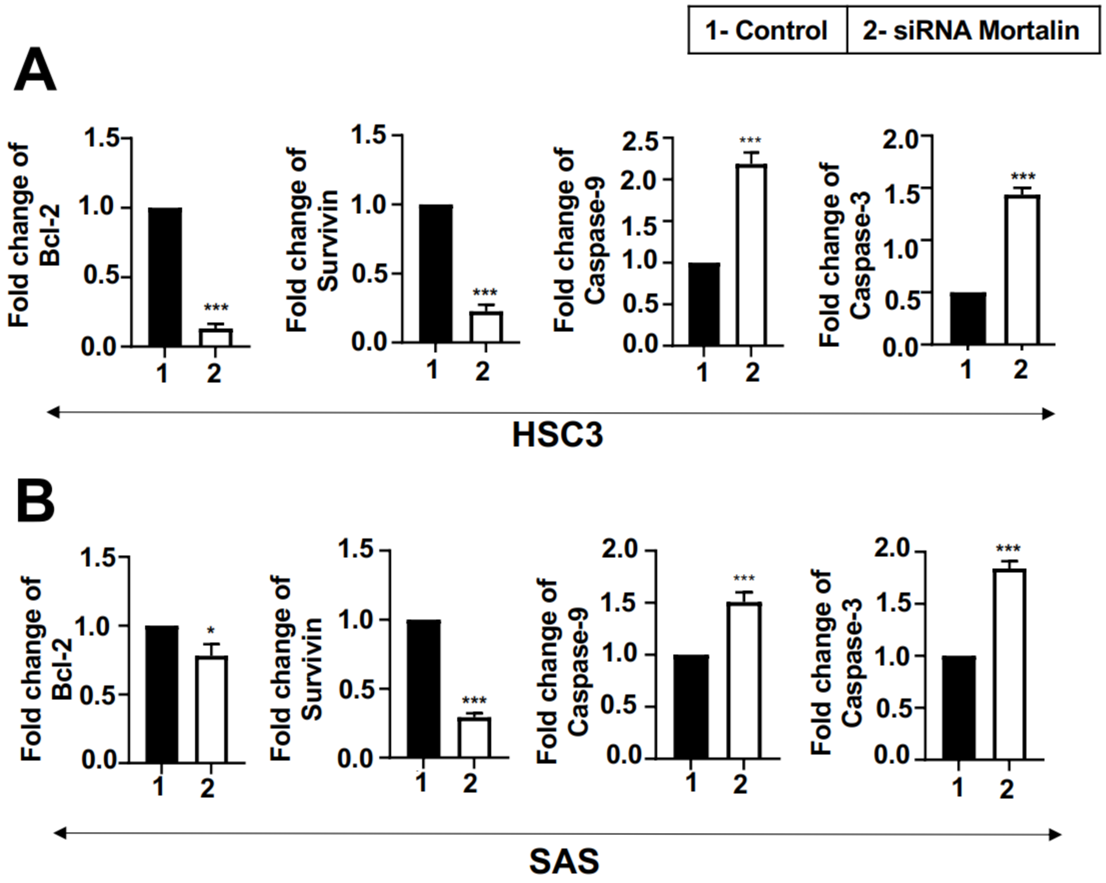

Figure S5. Supplementary to Figure 5C: Quantitative graphs representing the expression of proteins involved in apoptosis in mortalin knockdown HSC3 and SAS cells. *p*-values < 0.001 (***highly significant), and <0.05 (*significant).

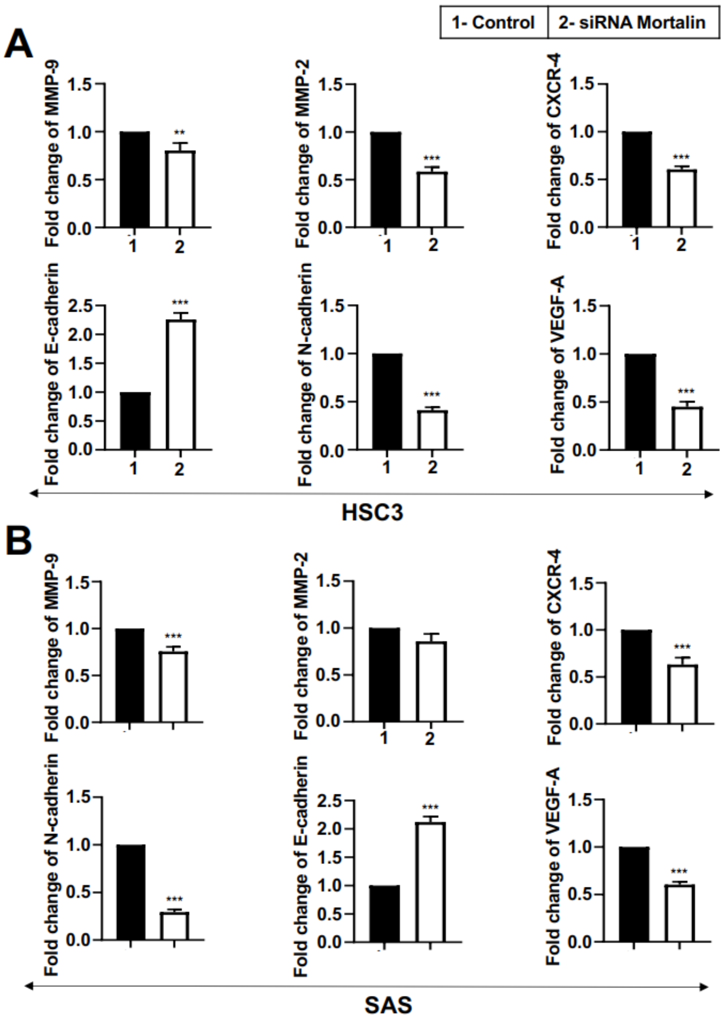

Figure S6 Supplementary to Figures 6A & 6B: Quantitative graphs representing the expression of proteins involved in EMT, angiogenesis, migration and invasion in mortalin inactivated oral cancer cells. *p*-values < 0.001 (***highly significant), and <0.01 (**very significant).

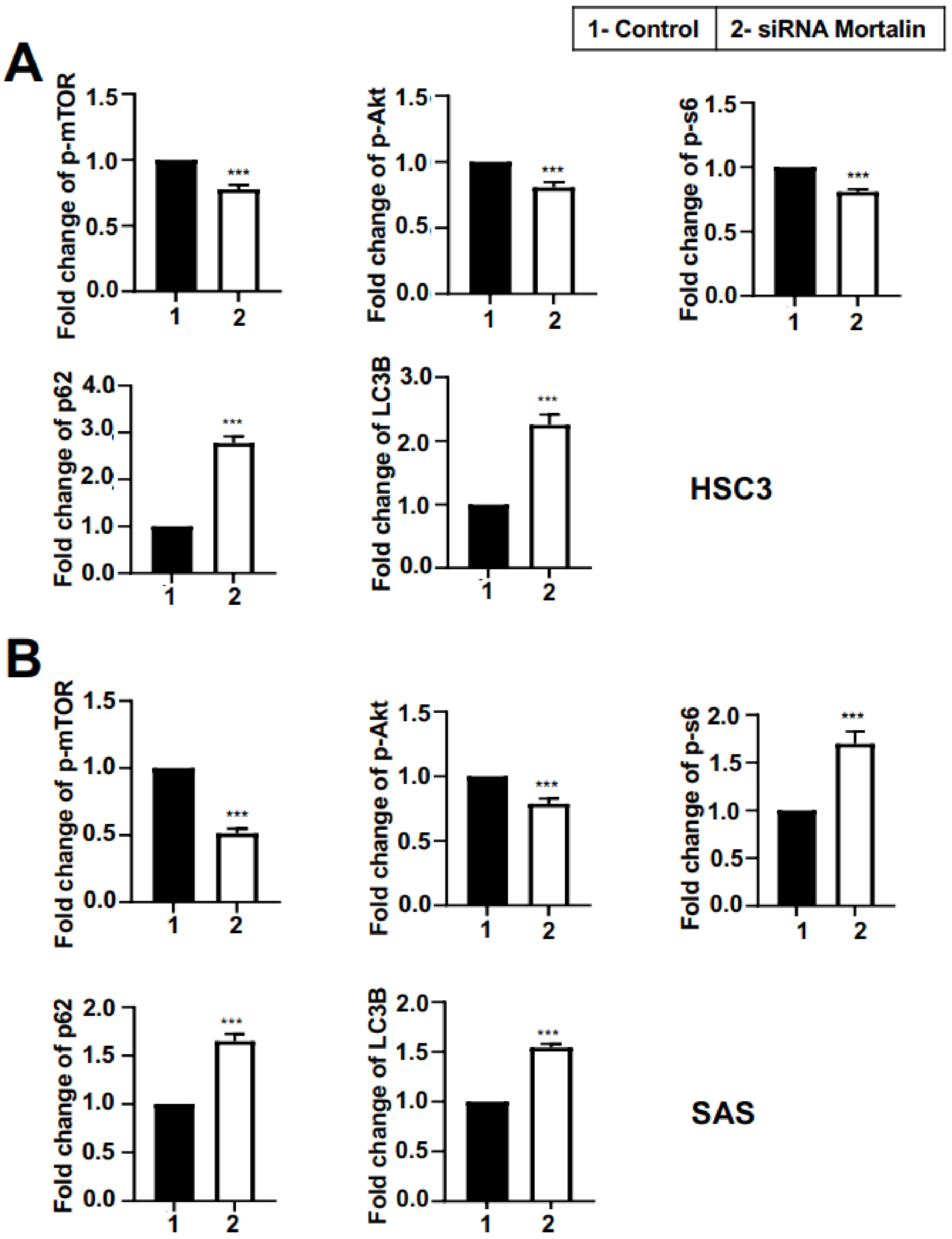

Figure S7. Supplementary to Figure 7: Quantitative representation of the expression of Akt signaling proteins and proteins involved in autophagy in mortalin knockdown HSC3 and SAS cells. *p*-values < 0.001 (***highly significant).
