# Supplementary material for: Mortalin Represents a Promising Therapeutic Target for Oral Cancers: Clinical Relevance and Experimental Evidence for the Activation of Akt/mTOR Signaling
Source: Cancers (Basel). 2025 Aug 30;17(17):2860. doi: 10.3390/cancers17172860 (PMC12427376; doi:10.3390/cancers17172860)
Supplement: Supplementary file 1 [file cancers-17-02860-s001.zip › cancers-3765961-supplementary Tables S1-S4.pdf]

Table S1: Patient specification of Oral Cancer Tissue Microarray samples

Cores- 208; Cases- 99; Row number-13;
Column number-16; Core Diameter (mm)-1;
Thickness (μm)-5

Pos	Age	Sex	Anatomic site	Pathology diagnosis	TNM	Grade	Stage	Type
A1	40	M	Upper jaw	Squamous cell carcinoma	T4N0M0	1	IV	Malignant
A2	40	M	Lower jaw	Squamous cell carcinoma	T4N0M0	1	IV	Malignant
A3	79	M	Cheek	Squamous cell carcinoma	T2N0M0	1	II	Malignant
A4	79	M	Cheek	Squamous cell carcinoma	T2N0M0	1	II	Malignant
A5	81	M	Lip	Squamous cell carcinoma	T2N0M0	1	II	Malignant
A6	81	M	Lip	Squamous cell carcinoma	T2N0M0	1	II	Malignant
A7	57	M	Tongue	Squamous cell carcinoma	T1N0M0	1	I	Malignant
A8	57	M	Tongue	Squamous cell carcinoma	T1N0M0	1	I	Malignant
A9	66	M	Tongue	Squamous cell carcinoma	T1N0M0	1	I	Malignant
A10	66	M	Tongue	Squamous cell carcinoma	T1N0M0	1	I	Malignant
A11	51	F	Cheek	Squamous cell carcinoma of left cheek	T4N0M0	1	IV	Malignant
A12	51	F	Cheek	Squamous cell carcinoma of left cheek	T4N0M0	1	IV	Malignant
A13	65	F	Cheek	Squamous cell carcinoma of right cheek	T2N0M0	1	II	Malignant
A14	65	F	Cheek	Squamous cell carcinoma of right cheek	T2N0M0	1	II	Malignant
A15	62	F	Cheek	Squamous cell carcinoma of left cheek	T1N0M0	1	I	Malignant
A16	62	F	Cheek	Squamous cell carcinoma of left cheek (sparse)	T1N0M0	1	I	Malignant
B1	46	F	Upper jaw	Squamous cell carcinoma of left upper jaw	T1N0M0	1	I	Malignant
B2	46	F	Upper jaw	Squamous cell carcinoma of left upper jaw	T1N0M0	1	I	Malignant
B3	68	M	Palate	Squamous cell carcinoma of right palate	T2N0M0	1	II	Malignant
B4	68	M	Palate	Squamous cell carcinoma of right palate	T2N0M0	1	II	Malignant
B5	56	F	Cheek	Squamous cell carcinoma of left cheek	T2N0M0	1	II	Malignant
B6	56	F	Cheek	Squamous cell carcinoma of left cheek	T2N0M0	1	II	Malignant
B7	60	M	Gingiva	Squamous cell carcinoma	T1N0M0	1	I	Malignant
B8	60	M	Gingiva	Squamous cell carcinoma	T1N0M0	1	I	Malignant
B9	55	M	Cheek	Squamous cell carcinoma of right cheek	T1N0M0	1	I	Malignant
B10	55	M	Cheek	Squamous cell carcinoma of right cheek	T1N0M0	1	I	Malignant

B11	78	M	Lip	Squamous cell carcinoma of lower lip	T1N0M0	1	I	Malignant
B12	78	M	Lip	Squamous cell carcinoma of lower lip	T1N0M0	1	I	Malignant
B13	41	F	Gingiva	Squamous cell carcinoma	T1N0M0	1	I	Malignant
B14	41	F	Gingiva	Squamous cell carcinoma	T1N0M0	1	I	Malignant
B15	46	F	Tongue	Squamous cell carcinoma	T1N0M0	1	I	Malignant
B16	46	F	Tongue	Squamous cell carcinoma	T1N0M0	1	I	Malignant
C1	78	F	Lip	Squamous cell carcinoma of lower lip	T1N0M0	1	I	Malignant
C2	78	F	Lip	Squamous cell carcinoma of lower lip	T1N0M0	1	I	Malignant
C3	70	F	Lip	Squamous cell carcinoma of lower lip	T1N0M0	1	I	Malignant
C4	70	F	Lip	Squamous cell carcinoma of lower lip	T1N0M0	1	I	Malignant
C5	35	F	Tongue	Squamous cell carcinoma	T2N0M0	1	II	Malignant
C6	35	F	Tongue	Squamous cell carcinoma	T2N0M0	1	II	Malignant
C7	39	F	Tongue	Squamous cell carcinoma	T1N0M0	1	I	Malignant
C8	39	F	Tongue	Squamous cell carcinoma (sparse)	T1N0M0	1	I	Malignant
C9	44	M	Gingiva	Squamous cell carcinoma	T2N0M0	1	II	Malignant
C10	44	M	Gingiva	Squamous cell carcinoma	T2N0M0	1	II	Malignant
C11	78	M	Tongue	Squamous cell carcinoma	T2N0M0	1	II	Malignant
C12	78	M	Tongue	Squamous cell carcinoma	T2N0M0	1	II	Malignant
C13	60	M	Gingiva	Squamous cell carcinoma of right lower gingiva (sparse)	T1N0M0	-	I	Malignant
C14	60	M	Gingiva	Squamous cell carcinoma of right lower gingiva	T1N0M0	1	I	Malignant
C15	54	F	Lip	Squamous cell carcinoma of lower lip	T1N0M0	1	I	Malignant
C16	54	F	Lip	Squamous cell carcinoma of lower lip	T1N0M0	1	I	Malignant
D1	75	F	Lip	Squamous cell carcinoma of lower lip	T1N0M0	1	I	Malignant
D2	75	F	Lip	Squamous cell carcinoma of lower lip	T1N0M0	1	I	Malignant
D3	70	M	Cheek	Squamous cell carcinoma	T2N0M0	1	II	Malignant
D4	70	M	Cheek	Squamous cell carcinoma	T2N0M0	1	II	Malignant
D5	73	M	Lip	Squamous cell carcinoma of lower lip	T1N0M0	1	I	Malignant
D6	73	M	Lip	Squamous cell carcinoma of lower lip	T1N0M0	1	I	Malignant
D7	48	F	Tongue	Squamous cell carcinoma	T2N0M0	1	II	Malignant
D8	48	F	Tongue	Squamous cell carcinoma	T2N0M0	1	II	Malignant
D9	60	M	Tongue	Squamous cell carcinoma	T1N0M0	1	I	Malignant
D10	60	M	Tongue	Squamous cell carcinoma	T1N0M0	1	I	Malignant

D11	86	F	Lip	Squamous cell carcinoma of lower lip	T4N0M0	1	IV	Malignant
D12	86	F	Lip	Squamous cell carcinoma of lower lip	T4N0M0	1	IV	Malignant
D13	55	M	Gingiva	Squamous cell carcinoma	T2N0M0	2	II	Malignant
D14	55	M	Gingiva	Squamous cell carcinoma	T2N0M0	2	II	Malignant
D15	57	M	Gingiva	Squamous cell carcinoma	T1N0M0	2	I	Malignant
D16	57	M	Gingiva	Squamous cell carcinoma	T1N0M0	2	I	Malignant
E1	41	M	Gingiva	Squamous cell carcinoma of left upper gingiva	T1N0M0	3	I	Malignant
E2	41	M	Gingiva	Squamous cell carcinoma of left upper gingiva	T1N0M0	3	I	Malignant
E3	47	M	Lower jaw	Squamous cell carcinoma of left lower jaw	T2N0M0	3	II	Malignant
E4	47	M	Lower jaw	Squamous cell carcinoma of left lower jaw	T2N0M0	3	II	Malignant
E5	45	F	Tongue	Squamous cell carcinoma	T3N0M0	3	III	Malignant
E6	45	F	Tongue	Squamous cell carcinoma	T3N0M0	3	III	Malignant
E7	48	F	Oral cavity	Mucinous adenocarcinoma	T2N0M0	2	II	Malignant
E8	48	F	Oral cavity	Mucinous adenocarcinoma	T2N0M0	2	II	Malignant
E9	60	M	Upper jaw	Adenocarcinoma	T1N0M0	1--2	I	Malignant
E10	60	M	Upper jaw	Adenocarcinoma	T1N0M0	1--2	I	Malignant
E11	8	F	Salivary gland	Adenocarcinoma	T2N0M0	*	II	Malignant
E12	8	F	Salivary gland	Adenocarcinoma	T2N0M0	*	II	Malignant
E13	28	M	Maxillary sinus	Adenocarcinoma of right maxillary sinus	T1N0M0	3	I	Malignant
E14	28	M	Maxillary sinus	Adenocarcinoma of right maxillary sinus	T1N0M0	3	I	Malignant
E15	71	M	Mouth	Mucoepidermoid carcinoma of left mouth floor	T1N0M0	1	I	Malignant
E16	71	M	Mouth	Mucoepidermoid carcinoma of left mouth floor	T1N0M0	1	I	Malignant
F1	50	F	Cheek	Mucoepidermoid carcinoma (sparse)	T2N0M0	1	II	Malignant
F2	50	F	Cheek	Mucoepidermoid carcinoma (sparse)	T2N0M0	1	II	Malignant
F3	57	M	Lip	Mucoepidermoid carcinoma of upper lip	T1N0M0	1--2	I	Malignant

F4	57	M	Lip	Mucoepidermoid carcinoma of upper lip	T1N0M0	1--2	I	Malignant
F5	48	F	Lower jaw	Mucoepidermoid carcinoma of right lower jaw	T1N0M0	2	I	Malignant
F6	48	F	Lower jaw	Mucoepidermoid carcinoma of right lower jaw	T1N0M0	2	I	Malignant
F7	57	M	Palate	Mucoepidermoid carcinoma of upper palate	T2N0M0	2	II	Malignant
F8	57	M	Palate	Mucoepidermoid carcinoma of upper palate	T2N0M0	2	II	Malignant
F9	32	F	Palate	Mucoepidermoid carcinoma (sparse)	T2N0M0	3	II	Malignant
F10	32	F	Palate	Mucoepidermoid carcinoma (sparse)	T2N0M0	3	II	Malignant
F11	55	M	Gingiva	Mucoepidermoid carcinoma of left upper gingiva	T1N0M0	3	I	Malignant
F12	55	M	Gingiva	Mucoepidermoid carcinoma of left upper gingiva	T1N0M0	3	I	Malignant
F13	50	M	Tongue	Mucoepidermoid carcinoma	T1N0M0	2--3	I	Malignant
F14	50	M	Tongue	Mucoepidermoid carcinoma	T1N0M0	2--3	I	Malignant
F15	53	M	Maxilla	Mucoepidermoid carcinoma of left maxilla	T2N0M0	3	II	Malignant
F16	53	M	Maxilla	Mucoepidermoid carcinoma of left maxilla	T2N0M0	3	II	Malignant
G1	32	F	Tongue	Mucoepidermoid carcinoma	T1N0M0	2--3	I	Malignant
G2	32	F	Tongue	Mucoepidermoid carcinoma	T1N0M0	2--3	I	Malignant
G3	79	F	Lip	Basal cell carcinoma	T2N0M0	-	II	Malignant
G4	79	F	Lip	Basal cell carcinoma	T2N0M0	-	II	Malignant
G5	60	F	Lip	Basal cell carcinoma of right lower lip	T1N0M0	-	I	Malignant
G6	60	F	Lip	Basal cell carcinoma of right lower lip	T1N0M0	-	I	Malignant
G7	58	M	Lymph node	Metastatic squamous cell carcinoma of neck	-	1	-	Metastasis
G8	58	M	Lymph node	Metastatic squamous cell carcinoma of neck	-	1	-	Metastasis
G9	55	M	Lymph node	Metastatic squamous cell carcinoma of neck	-	1--2	-	Metastasis
G10	55	M	Lymph node	Metastatic squamous cell carcinoma of neck	-	1--2	-	Metastasis

G11	40	F	Lymph node	Metastatic squamous cell carcinoma of left lower mandible	-	*	-	Metastasis
G12	40	F	Lymph node	Metastatic squamous cell carcinoma of left lower mandible	-	3	-	Metastasis
G13	52	F	Lymph node	Metastatic acinic cell carcinoma of neck	-	-	-	Metastasis
G14	52	F	Lymph node	Metastatic acinic cell carcinoma of neck	-	-	-	Metastasis
G15	11	M	Mandible	Adamantinoma	-	-	-	Benign
G16	11	M	Mandible	Adamantinoma	-	-	-	Benign
H1	62	M	Mandible	Adamantinoma	-	-	-	Benign
H2	62	M	Mandible	Adamantinoma	-	-	-	Benign
H3	28	M	Lower jaw	Adamantinoma of left lower jaw	-	-	-	Benign
H4	28	M	Lower jaw	Adamantinoma of left lower jaw	-	-	-	Benign
H5	38	M	Maxilla	Adamantinoma	-	-	-	Benign
H6	38	M	Maxilla	Adamantinoma	-	-	-	Benign
H7	37	F	Mandible	Adamantinoma	-	-	-	Benign
H8	37	F	Mandible	Adamantinoma	-	-	-	Benign
H9	34	M	Mandible	Adamantinoma with necrosis of left mandible (sparse)	-	-	-	Benign
H10	34	M	Mandible	Adamantinoma of left mandible	-	-	-	Benign
H11	40	M	Lower jaw	Adamantinoma	-	-	-	Benign
H12	40	M	Lower jaw	Adamantinoma	-	-	-	Benign
H13	47	F	Mandible	Adamantinoma	-	-	-	Benign
H14	47	F	Mandible	Adamantinoma	-	-	-	Benign
H15	70	F	Mandible	Adamantinoma of right mandible	-	-	-	Benign
H16	70	F	Mandible	Adamantinoma of right mandible	-	-	-	Benign

I1	51	M	Mandible	Adamantinoma of right mandible	-	-	-	Benign
I2	51	M	Mandible	Adamantinoma of right mandible	-	*	-	Benign
I3	60	M	Gingiva	Ulcer of right lower gingiva mucosa of No.45	-	-	-	Ulcer
I4	60	M	Gingiva	Ulcer of right lower gingiva mucosa of No.46	-	-	-	Ulcer
I5	76	M	Palate	Cyst of right upper palate	-	*	-	Cyst
I6	76	M	Palate	Cyst of right upper palate	-	-	-	Cyst
I7	22	F	Mandible	Cyst of left mandible	-	*	-	Cyst
I8	22	F	Mandible	Cyst of left mandible	-	*	-	Cyst
I9	86	M	Lip	Mild hyperplasia of squamous epithelium of right upper lip	-	-	-	Hyperplas ia
I10	86	M	Lip	Mild hyperplasia of squamous epithelium of right upper lip	-	-	-	Hyperplas ia
I11	48	F	Tongue	Mild hyperplasia of squamous epithelium of No.55	-	-	-	Hyperplas ia
I12	48	F	Tongue	Mild hyperplasia of squamous epithelium of No.56	-	-	-	Hyperplas ia
I13	46	M	Tongue	Mild hyperplasia of squamous epithelium (sparse)	-	-	-	Hyperplas ia
I14	46	M	Tongue	Mild hyperplasia of squamous epithelium (sparse)	-	-	-	Hyperplas ia
I15	40	M	Lip	Mild hyperplasia of squamous epithelium of right lower lip	-	*	-	Hyperplas ia
I16	40	M	Lip	Mild hyperplasia of squamous epithelium of right lower lip	-	-	-	Hyperplas ia
J1	53	M	Cheek	Mild-moderate atypical hyperplasia of left cheek	-	-	-	Hyperplas ia
J2	53	M	Cheek	Mild-moderate atypical hyperplasia of left cheek	-	-	-	Hyperplas ia
J3	62	F	Cheek	Chronic Inflammation of mucosa squamous epithelium	-	-	-	Inflammat ion
J4	62	F	Cheek	Chronic Inflammation of mucosa squamous epithelium	-	-	-	Inflammat ion
J5	58	F	Salivary gland	Chronic Inflammation of right palate	-	-	-	Inflammat ion

J6	58	F	Salivary gland	Chronic Inflammation of right palate	-	-	-	Inflammat ion
J7	32	M	Salivary gland	Chronic Inflammation of right submandibular gland (sparse)	-	-	-	Inflammat ion
J8	32	M	Salivary gland	Chronic Inflammation of right submandibular gland	-	-	-	Inflammat ion
J9	63	M	Cheek	Chronic Inflammation of mucosa with focal epithelium hyperplasia of left cheek	-	-	-	Inflammat ion
J10	63	M	Cheek	Chronic Inflammation of mucosa with focal epithelium hyperplasia of left cheek	-	-	-	Inflammat ion
J11	63	M	Lower jaw	Chronic Inflammation of salivary gland of right lower jaw	-	-	-	Inflammat ion
J12	63	M	Lower jaw	Chronic Inflammation of salivary gland of right lower jaw	-	-	-	Inflammat ion
J13	45	F	Tongue	Chronic Inflammation with mild hyperplasia of squamous epithelium of No.69	-	-	-	Inflammat ion
J14	45	F	Tongue	Chronic Inflammation with mild hyperplasia of squamous epithelium of No.70	-	-	-	Inflammat ion
J15	63	M	Salivary gland	Chronic Inflammation of right submandibular gland	-	-	-	Inflammat ion
J16	63	M	Salivary gland	Chronic Inflammation of right submandibular gland	-	-	-	Inflammat ion
K1	32	F	Upper jaw	Chronic mucositis of right upper jaw	-	-	-	Inflammat ion
K2	32	F	Upper jaw	Chronic mucositis of right upper jaw	-	-	-	Inflammat ion
K3	75	F	Lip	Chronic Inflammation of skin of lower lip of No.49	-	-	-	Inflammat ion
K4	75	F	Lip	Chronic Inflammation of skin of lower lip of No.50	-	-	-	Inflammat ion
K5	52	F	Tongue	Chronic Inflammation	-	*	-	Inflammat ion
K6	52	F	Tongue	Chronic Inflammation	-	*	-	Inflammat ion
K7	15	M	Salivary gland	Chronic Inflammation	-	-	-	Inflammat ion
K8	15	M	Salivary gland	Chronic Inflammation	-	-	-	Inflammat ion

K9	48	F	Salivary gland	Adjacent normal salivary gland tissue of No.55	-	-	-	NAT
K10	48	F	Salivary gland	Adjacent normal salivary gland tissue of No.56	-	-	-	NAT
K11	37	M	Salivary gland	Adjacent normal salivary gland tissue	-	-	-	NAT
K12	37	M	Salivary gland	Adjacent normal salivary gland tissue	-	-	-	NAT
K13	63	M	Salivary gland	Adjacent normal salivary gland tissue	-	-	-	NAT
K14	63	M	Salivary gland	Adjacent normal salivary gland tissue	-	-	-	NAT
K15	45	M	Salivary gland	Adjacent normal salivary gland tissue	-	-	-	NAT
K16	45	M	Salivary gland	Adjacent normal salivary gland tissue	-	-	-	NAT
L1	45	M	Salivary gland	Cancer adjacent salivary gland tissue	-	-	-	AT
L2	45	M	Salivary gland	Cancer adjacent salivary gland tissue	-	-	-	AT
L3	47	M	Salivary gland	Adjacent normal salivary gland tissue	-	-	-	NAT
L4	47	M	Salivary gland	Adjacent normal salivary gland tissue	-	-	-	NAT
L5	13	F	Salivary gland	Adjacent normal salivary gland tissue	-	-	-	NAT
L6	13	F	Salivary gland	Adjacent normal salivary gland tissue	-	-	-	NAT
L7	33	F	Salivary gland	Adjacent normal salivary gland tissue (sparse)	-	-	-	NAT
L8	33	F	Salivary gland	Adjacent normal salivary gland tissue	-	-	-	NAT
L9	39	F	Tongue	Adjacent normal tongue tissue of No.39	-	-	-	NAT
L10	39	F	Tongue	Adjacent normal tongue tissue of No.40	-	-	-	NAT
L11	51	F	Tongue	Adjacent normal tongue tissue	-	-	-	NAT
L12	51	F	Tongue	Adjacent normal tongue tissue	-	-	-	NAT
L13	43	M	Tongue	Tongue tissue	-	-	-	Normal
L14	43	M	Tongue	Tongue tissue	-	-	-	Normal
L15	42	F	Tongue	Tongue tissue	-	-	-	Normal
L16	42	F	Tongue	Tongue tissue	-	-	-	Normal

M1	21	F	Tongue	Tongue tissue	-	-	-	Normal
M2	21	F	Tongue	Tongue tissue	-	-	-	Normal
M3	16	M	Tongue	Tongue tissue	-	-	-	Normal
M4	16	M	Tongue	Tongue tissue	-	-	-	Normal
M5	48	M	Tongue	Tongue tissue	-	-	-	Normal
M6	48	M	Tongue	Tongue tissue	-	-	-	Normal
M7	22	M	Salivary gland	Salivary gland tissue	-	-	-	Normal
M8	22	M	Salivary gland	Salivary gland tissue	-	-	-	Normal
M9	50	M	Salivary gland	Salivary gland tissue	-	-	-	Normal
M10	50	M	Salivary gland	Salivary gland tissue	-	-	-	Normal
M11	15	F	Tongue	Tongue tissue	-	-	-	Normal
M12	15	F	Tongue	Tongue tissue	-	-	-	Normal
M13	45	M	Salivary gland	Salivary gland tissue	-	-	-	Normal
M14	45	M	Salivary gland	Salivary gland tissue	-	-	-	Normal
M15	38	F	Salivary gland	Salivary gland tissue	-	-	-	Normal
M16	38	F	Salivary gland	Salivary gland tissue	-	-	-	Normal
-	58	M	Skin	Malignant melanoma (tissue marker)		-		Malignant

Table S2: Scoring method for IHC

<table>
<tr><td>Score (P)</td><td>0</td><td>1+</td><td>2+</td><td>3+</td><td>4+</td></tr>
<tr><td>Positive cells</td><td><10%</td><td>10-25%</td><td>25-50%</td><td>50-75%</td><td>>75%</td></tr>
<tr><td>Score (I)</td><td>1</td><td>2</td><td>3</td><td colspan="2" rowspan="2">Total expression score

Q= P*I</td></tr>
<tr><td>Intensity of Stain</td><td>Weak</td><td>Moderate</td><td>Strong</td></tr>
</table>

Table S3: Details of head and neck cancer patient samples collected from Northeast India.

Sample No.	Age	Sex	Anatomic Site
N1	45	F	Head & Neck
N2	46	M	Alveolus (left)
N3	50	F	Buccal mucosa- lower lip
N4	77	M	Growth lower alveolus
T1	51	F	Buccal mucosa
T2	70	F	Buccal mucosa of tongue
T3	58	M	Buccal mucosa (right) + retromolar trigone
T4	50	F	Buccal mucosa-lower lip
T5	41	F	Buccal mucosa
T6	45	F	Upper Alveolus (left)
T7	50	F	Buccal mucosa (left)
T8	62	M	AEF
T9	62	M	Buccal mucosa of tongue
T10	42	M	Thyroid
T11	63	M	Lower Alveolus (right)
T12	56	M	Buccal mucosa (left)
T13	47	M	Maxilla (right)
T14	62	M	Buccal mucosa of tongue
T15	54	M	Thyroid
T16	70	M	Left Alveolus SNN
T17	56	M	PFS
T18	65	M	Buccal mucosa (right)
T19	57	M	Buccal mucosa
T20	42	M	Tongue
T21	65	M	Buccal mucosa (right)

Table S4: Details of primary and secondary antibodies.

Name	RRID*
Anti-GAPDH antibody, 2118S, Cell Signaling Technology, USA	RRID:AB_561053
Anti-p53 antibody, 2524S, Cell Signaling Technology, USA	RRID:AB_331743
Anti-p27 antibody, 3686, Cell Signaling Technology, USA	RRID:AB_2077850
Anti-p21 antibody, 2947, Cell Signaling Technology, USA	RRID:AB_823586
Anti-phospho-wee1 (Ser642) 4910, Cell Signaling Technology, USA	RRID:AB_2215870
Anti-cyclin D1 antibody 2978, Cell Signaling Technology, USA	RRID:AB_2259616
Anti-cyclin B1 antibody 12231, Cell Signaling Technology, USA	RRID:AB_2783553
Anti-cyclin E2 antibody 4132, Cell Signaling Technology, USA	RRID:AB_2071197
Anti-Caspase3 antibody BB-AB0243S, Bio Bharati Life Science, India	-
Anti-Caspase9 antibody BB-AB0245S, Bio Bharati Life Science, India	-
Anti-Bcl-2 antibody, #BB-AB0230, Bio Bharati Life Science, India	-
Anti-COX-2 antibody 12282P, Cell Signaling Technology, USA	RRID:AB_2571729
Anti-Survivin antibody 2808BC, Cell Signaling Technology, USA	RRID:AB_2063948
Anti-MMP-2 antibody 4022, Cell Signaling Technology, USA	RRID:AB_2266622
Anti-MMP-9 antibody 13667P, Cell Signaling Technology, USA	RRID:AB_2798289
Anti-CXCR4 antibody ab124824, abcam, Cambridge, USA	RRID: AB_10975635
Anti-E-cadherin antibody 3195S, Cell Signaling Technology, USA	RRID:AB_2291471
Anti-N-cadherin antibody 13116S, Cell Signaling Technology, USA	RRID:AB_2687616
Anti-VEGF-A antibody ab46154, abcam, Cambridge, USA	RRID:AB_2212642
Anti-phospho- Akt (Ser473) antibody, 4060S, Cell Signaling Technology, USA	RRID:AB_2315049
Anti- Akt1 antibody 2938S, Cell Signaling Technology, USA	RRID:AB_915788
Anti-phospho- mTOR (Ser2448) antibody 5536T, Cell Signaling Technology, USA	RRID:AB_10691552
Anti-mTOR antibody 2983T, Cell Signaling Technology, USA	RRID:AB_2105622
Anti-phospho-S6 ribosomal protein (Ser235/236) antibody 4858T, Cell Signaling Technology, USA	RRID:AB_916156

Anti-S6 ribosomal protein antibody 2317S, Cell Signaling Technology, USA	RRID: AB_2238583
Anti-LC3B antibody 2775S, Cell Signaling Technology, USA	RRID:AB_915950
Anti-SQSTM1/p62, 8025S, Cell Signaling Technology, USA	RRID:AB_10859911
Anti-mouse secondary antibody ab97040, abcam, Cambridge, USA	RRID:AB_10698223
Anti-rabbit secondary antibody ab97080, abcam, Cambridge, USA	RRID:AB_2210028

***Research Resource Identifier (RRID).**
